# Depletion of *Sorcs3* Activates Totipotency in Mouse Embryonic Stem Cells by Modulating Key Signaling Pathways

**DOI:** 10.1002/advs.202509151

**Published:** 2025-11-03

**Authors:** Wenhao Zhang, Xinyu Mao, Yu He, Qingshen Jia, Yiding Zhao, Xiaomeng Dai, Xiaoyan Li, Shengyi Sun, Xiaoyan Sheng, Dan Ding, Yuan Shi, Qian Gao, Ling Shuai

**Affiliations:** ^1^ State Key Laboratory of Medicinal Chemical Biology College of Pharmacy Nankai University, Animal Resources Center and Reproductive Regulation, and Institute of Transplantation Medicine Nankai University Tianjin 300350 China; ^2^ Department of Neonatology Children's Hospital of Chongqing Medical University, National Clinical Research Center for Child Health and Disorders, Ministry of Education Key Laboratory of Child Development and Disorders, China International Science and Technology Cooperation Base of Child Development and Critical Disorders Chongqing Key Laboratory of Child Rare Diseases in Infection and Immunity Chongqing 400014 China

**Keywords:** ESCs, signaling pathway, Sorcs3, Tfap2c, totipotency

## Abstract

Totipotency represents the greatest potential to yield an entire individual alongside its associated extraembryonic tissues, albeit transiently. Nevertheless, achieving sustainable totipotent stem cells remains an intriguing yet challenging endeavor. Here, it is reported that *Sorcs3* depletion in murine embryonic stem cells (ESCs) enables robust differentiation into both embryonic and extraembryonic lineages, resulting in a totipotent‐like state. Notably, *Sorcs3* knockout (SKO)‐ESCs can efficiently self‐assemble into typical blastocyst‐like structures, offering a versatile model for studying early embryonic development. Comprehensive analyses reveal that totipotency in SKO‐ESCs is related to *Tfap2c* gene activation. Deletion of *Tfap2c* significantly reduces the developmental potential of SKO‐ESCs across all the examined phenotypes, underscoring its critical role. Furthermore, single‐cell transcriptome analysis of SKO‐ESCs reveals that inhibition of the TGF‐β, PI3K‐AKT, and lysosome pathways drives totipotency activation, which is validated by the introduction of corresponding inhibitors into wild‐type ESC cultures. Together, the findings facilitate the establishment of totipotent stem cells in a defined medium and provide a universal platform for studying totipotency.

## Graphical abstract

In this article, Shuai and colleagues found that depletion of *Sorcs3* could activate totipotency in mESCs, promising bidirectional differentiation potential to embryonic and extraembryonic lineages. The underlying mechanism might be activation of *Tfap2c* and repression of TGF‐β, PI3K‐AKT, and lysosome pathways. These findings facilitate understanding of totipotency acquisition in a brand‐new view.

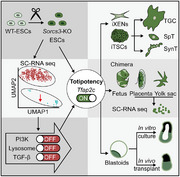

## 1 Introduction

The preimplantation development of murine embryos involves an ordered shift in cell potency, during which totipotent blastomeres transition to segregated cells after the 4‐cell stage. After this stage, embryos begin to form inner cell masses (ICMs) and trophectoderms (TEs).^[^ ^1^ ^]^ It is difficult to achieve natural switches between these groups of cells because of their lineage restriction. Early blastomeres are totipotent but lack self‐renewal ability,^[^ ^2^ ^]^ making them unsuitable for studying the acquisition and maintenance of totipotency. Embryonic stem cells (ESCs), extraembryonic endoderm cells (XENs) and trophoblast stem cells (TSCs) can be derived from blastocysts separately, preserving most of the epiblast (EPI), primitive endoderm (PrE), and TE natures and proliferative abilities, respectively.^[^ ^3^, ^4^, ^5^ ^]^ ESCs are thus ideal cell models for studying pluripotency expansion. Establishing and maintaining totipotent stem cells with features resembling early blastomeres is both important and difficult. Recently, a few totipotent‐like stem cells were reported.^[^ ^6^, ^7^, ^8^ ^]^ These studies focused mainly on how epigenetic changes activated certain 2‐cell embryo (2C)‐specific genes. However, whether these 2C‐like stem cells (through the activation of representative 2C genes) possess totipotent developmental potential for use with embryonic and extraembryonic tissues in rigorous chimera assays and blastoid‐formation experiments remains unknown. Among them, only TBLCs from the Du group were reported to have these competencies with high efficiency simultaneously.^[^ ^9^ ^]^ It would be interesting to determine whether the acquisition of definite totipotency can be achieved through the modulation of a single gene and its related pathways.

Our group has been engaged in genetic screening using haploid ESCs (haESCs) for years. We revealed many candidates when identifying genes restricted to totipotency using murine haESCs, including *Sorcs3*. SORCS3, sortilin‐related VPS10 domain‐containing receptor 3, belongs to the vacuolar protein sorting 10 protein (VPS10p) receptor family. VPS10p domain receptors are a unique class of sorting receptors that direct the intracellular transport of target proteins between the cell surface, endosomes, Golgi bodies, and lysosomes in mammalian cells and possess receptor internalization abilities.^[^ ^10^ ^]^ Given that *Sorcs3* is important for internalization, examining its effect on differentiation capacity is highly important.

Here, we investigate whether the deletion of *Sorcs3* enables mouse ESCs (mESCs) to enter extraembryonic cell fates. We also assess the differentiation potential of *Sorcs3* knockout (SKO)‐ESCs through chimera assays and blastoid formation experiments. Next, we comprehensively compare SKO‐ESCs with wild‐type (WT)‐ESCs at the single‐cell transcriptome and developmental levels to elucidate the mechanisms underlying totipotency acquisition.

## 2 Results

### 2.1 *Sorcs3*‐KO Allows mESCs to Differentiate into Extraembryonic Lineages

**Figure 1:**
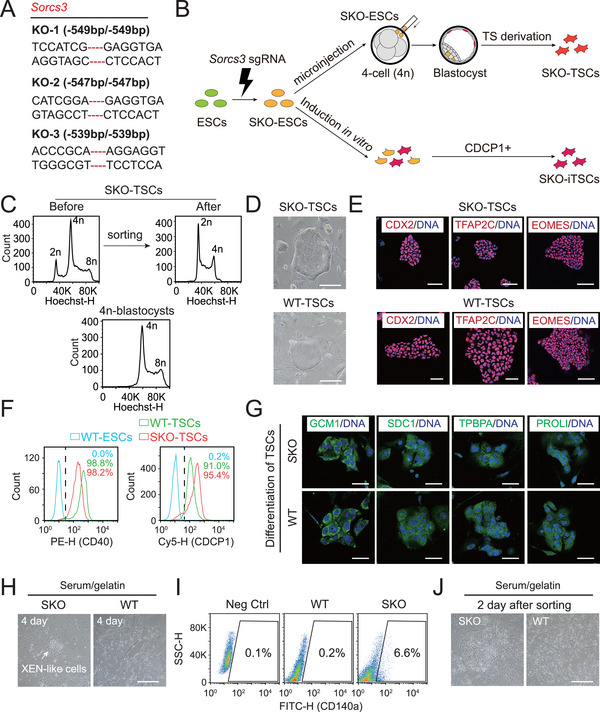
Extraembryonic potential of differentiated SKO‐ESCs. A) DNA sequencing validation of SKO subclones. B) Strategy for generating *Sorcs3*‐KO trophoblast stem cells (SKO‐TSCs) via tetraploid complementation and *Sorcs3*‐KO induced TSCs (SKO‐iTSCs) through in vitro differentiation. C) DNA content analysis of SKO‐TSCs before and after sorting. Tetraploid blastocysts served as controls. D) Morphology of SKO‐TSC and WT‐TSC colonies. Scale bar: 100 µm. E) Immunofluorescence (IF) of TSC markers (CDX2, TFAP2C, and EOMES) in SKO‐TSCs and WT‐TSCs. Hoechst 33342 (blue) is utilized to label DNA. Scale bar: 50 µm. F) FACS analysis of the CD40^+^ and CDCP1^+^ populations among SKO‐TSCs and WT‐TSCs. SKO‐ESCs served as negative controls. G) IF of differentiated trophoblast markers (GCM1, SDC1, TPBPA, and PROLIFERIN) in terminally differentiated cultures of SKO‐TSCs and WT‐TSCs. Hoechst 33342 (blue) is utilized to label DNA. Scale bar: 50 µm. H) Spontaneous differentiation of SKO‐ESCs and WT‐ESCs on Day 4 as determined by bright field (BF) microscopy. Scale bar: 100 µm. I) Cell sorting with a XEN‐specific antibody (CD140a) in differentiated cultures of SKO‐ESCs and WT‐ESCs, with WT‐ESCs as negative controls. J) BF images of the sorted cells 2 days after CD140a enrichment. Only the SKO group contained numerous XEN‐like cells. Scale bar: 100 µm.

To determine the role of *Sorcs3* in preimplantation development, we measured its expression levels at different stages by immunostaining. We found that SORCS3 was very active at the morula stage (Figure S1A, Supporting Information). It is widely believed that mouse blastomeres exhibit plasticity and segregate to distinct cell fates in this stage.^[^ ^11^, ^12^ ^]^ To investigate whether *Sorcs3* knockout (SKO) enhances cell plasticity, we designed specific Cas9‐green fluorescent protein (GFP) guide RNAs (gRNAs) to knockout *Sorcs3* expression in WT‐ESCs (Figure S1B, Supporting Information). After electroporation and subclone selection, three KO (‐/‐) lines were obtained and further confirmed by PCR, DNA sequencing, and immunostaining (**Figure** **1**A; Figure S1C,D, Supporting Information). We utilized these SKO‐ESC lines for subsequent experiments. To assess the extraembryonic differentiation potential of SKO‐ESCs, we adapted two different strategies to induce trophoblast cell differentiation (Figure 1B).

First, we adapted a chimera experiment to induce trophoblast fate. We microinjected SKO‐ESCs into tetraploid embryos to construct chimeric embryos, which were further seeded in TSC medium to generate TSCs at the blastocyst stage. Chimeric blastocyst‐derived TSCs were enriched for diploid ESCs (SKO‐ESC donors, diploid; recipient embryo, tetraploid), from which SKO‐TSCs were obtained (Figure 1C). These results indicated that SKO‐ESCs were capable of differentiating into the TE lineage during the course of embryonic development. SKO‐TSCs not only exhibited a colony morphology typical of TSCs but also expressed the trophoblast‐specific markers CDX2, TFAP2C, and EOMES, similar to WT‐TSCs (a cell line derived from a WT blastocyst) (Figure 1D,E). In addition, the SKO‐TSCs maintained high proportions of CD40‐positive (98.2%) and CDCP1‐positive (95.4%) cells (two TSC‐specific flow cytometric sorting antibodies) in long‐term culture according to fluorescence‐activated cell sorting (FACS) analysis, with WT‐ESCs and WT‐TSCs as negative and positive controls, respectively (Figure 1F). Next, we induced spontaneous differentiation of the SKO‐TSCs by withdrawing the feeder layers and growth factors (FGF4 and heparin). The respective differentiated cell cultures were positive for GCM1, SDC1 (a syncytiotrophoblast‐specific marker), TPBPA (a spongiotrophoblast‐specific marker), and PROLIFERIN (a trophoblast giant cell‐specific marker), indicating that SKO‐TSCs had further potential to differentiate into terminal trophoblast lineages (Figure 1G). In parallel with spontaneous differentiation, DNA content analysis of the differentiated cells on Days 0, 4, and 8 revealed that 8n polyploidy increased sharply, indicating the emergence of differentiating trophoblast giant cells (Figure S1E, Supporting Information). However, these results could not be achieved in WT‐ESCs.

In the second strategy, we cultured SKO‐ESCs in TSC medium to induce TSC differentiation in vitro directly. After 12 days of induction, the percentage of CDCP1‐positive (+) cells among the *Sorcs3*‐KO ESCs increased gradually, whereas that among the WT‐ESCs did not (Figure S1F, Supporting Information). Typically, induced TSCs (iTSCs) could be established in the SKO group after CDCP1^+^ cell sorting and showed typical TSC morphology, whereas the WT‐ESC group lacked this competency (Figure S1G, Supporting Information). In addition to assessing trophectoderm differentiation, we conducted an independent spontaneous differentiation assay under serum/gelatin conditions (without growth factors) to induce PrE lineage (XEN‐like cell) differentiation using SKO‐ESCs and WT‐ESCs separately. Four days after differentiation induction, XEN‐like colonies were observed in the SKO group only, with 6.6% CD140a^+^ (a PrE‐specific antibody) cells, whereas no such positive cells were detected in the WT group (Figure 1H,I). Two days after cell sorting, typical XEN‐like cells were exclusively observed in the SKO group (Figure 1J), demonstrating that compared with WT‐ESCs, SKO‐ESCs indeed exhibited enhanced plasticity to the PrE lineage.

### 2.2 SKO‐ESCs Exhibit Totipotent Developmental Potential in Chimera Assays

**Figure 2:**
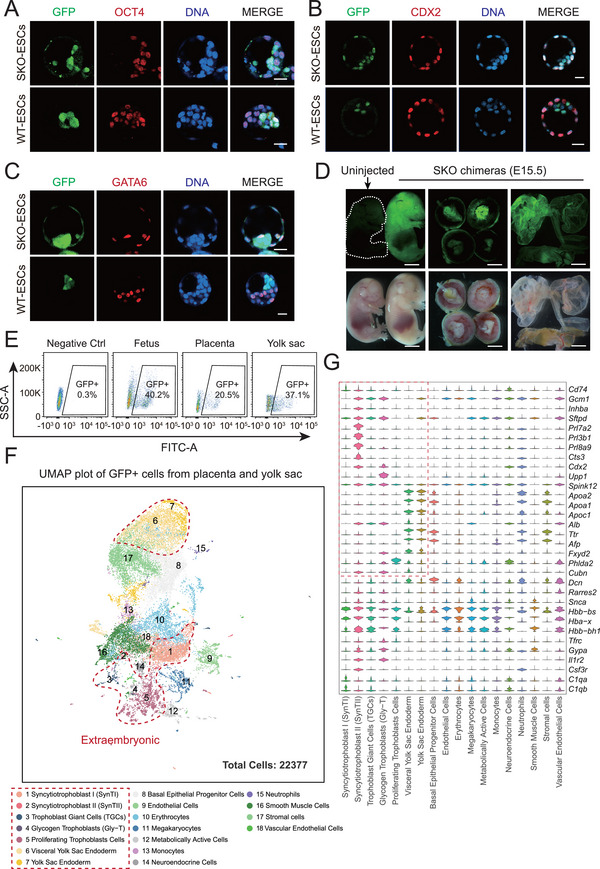
Totipotency developmental potential of *Sorcs3*‐KO ESCs. A) IF of OCT4 (red) in chimeric blastocysts injected with SKO‐ESCs (green) and WT‐ESCs (green), and Hoechst 33342 (blue) is utilized to label nuclei. Scale bar, 50 µm. B) IF of CDX2 (red) in chimeric blastocysts injected with SKO‐ESCs (green) and WT‐ESCs (green), and Hoechst 33342 (blue) is utilized to label nuclei. Scale bar, 50 µm. C) IF of GATA6 (red) in chimeric blastocysts injected with SKO‐ESCs (green) and WT‐ESCs (green), and Hoechst 33342 (blue) is utilized to label nuclei. Scale bar, 50 µm. D) E15.5 chimeric embryos (fetuses, placentas and yolk sacs) derived from GFP‐labeled SKO‐ESCs, with a noninjected fetus as a negative control. Scale bar: 5 mm. E) FACS analysis of GFP^+^ cells among the following chimeric tissues: fetus (40.2%), placenta (20.5%), and yolk sac (37.1%). Noninjected embryos served as negative controls (*n* = 3). F) UMAP plot of all clusters of GFP^+^ cells in the chimeric placenta and yolk sac. The extraembryonic lineages are marked. G) Violin plots displaying the relative distributions of the expression of specific marker genes in each cluster. The red dotted line highlights the extraembryonic cell lineages.

Given that compared with WT‐ESCs, SKO‐ESCs presented expanded pluripotency, determining whether they were totipotent during more refined and stringent in vivo development was necessary. We microinjected GFP‐labeled SKO‐ESCs and GFP‐labeled WT‐ESCs into mouse 4‐cell embryos separately to construct chimeras. Immunostaining analysis of chimeric blastocysts revealed that SKO‐ESCs could easily contribute to EPI (OCT4^+^), TE (CDX2^+^), and PrE (GATA6^+^) tissues, whereas WT‐ESCs were only able to integrate into the EPI region (**Figure** **2**A–C). Herein, compared with WT‐ESCs, SKO‐ESCs exhibited a totipotent‐like state with embryonic and extraembryonic bidirectional potential. A stricter assessment of totipotency was subsequently performed. The reconstructed embryos were transferred to the uteri of pseudopregnant mice for further development. Interestingly, compared with injected control cells, SKO‐ESCs contributed markedly to the embryonic and extraembryonic tissues of embryonic day (E)6.5, E12.5, and E15.5 chimeric embryos. However, the WT‐ESC group did not have this ability (Figure 2D; Figure S2A–D; Table S1, Supporting Information). FACS analysis further confirmed that GFP‐labeled SKO‐ESCs contributed 40.2% of the chimeric fetus, 20.5% of the chimeric placenta, and 37.1% of the chimeric yolk sac at E15.5 (Figure 2E). Next, we sorted the GFP^+^ cells from the chimeric placenta and the chimeric yolk sac for single‐cell RNA (scRNA)‐seq. By integrating graph‐based clustering with well‐known marker genes, we annotated 18 distinct clusters among all 22377 cells obtained that remained after computational quality control (Figure 2F). These clusters encompassed 7 extraembryonic lineages: syncytiotrophoblast I (SynTI), syncytiotrophoblast II (SynTII), trophoblast giant cells (TGCs), glycogen trophoblasts (GlyT), proliferating trophoblast cells, and two types of yolk sac cells. Additionally, we defined 11 embryonic lineages originating from SKO‐ESCs (Figure 2F,G). Thus, these data proved the robust bidirectional developmental potential of SKO‐ESCs in chimeric mice. In another parallel experiment, analysis of immunofluorescence sections from the SKO‐ESC‐derived E12.5 placenta confirmed that SKO‐ESCs contributed to functional placental tissues (TPBPA/GFP‐, TFAP2C/GFP‐, GCM1/GFP‐, and PROLIFERIN/GFP‐double positive separately) (Figure S2E, Supporting Information). All of the above chimeric assays were validated in at least two SKO cell lines. These results demonstrated that SKO‐ESCs indeed had totipotent developmental potential in these strict chimera assays in vivo.

### 2.3 SKO‐ESCs can Efficiently Produce Good‐Quality Blastoids

**Figure 3:**
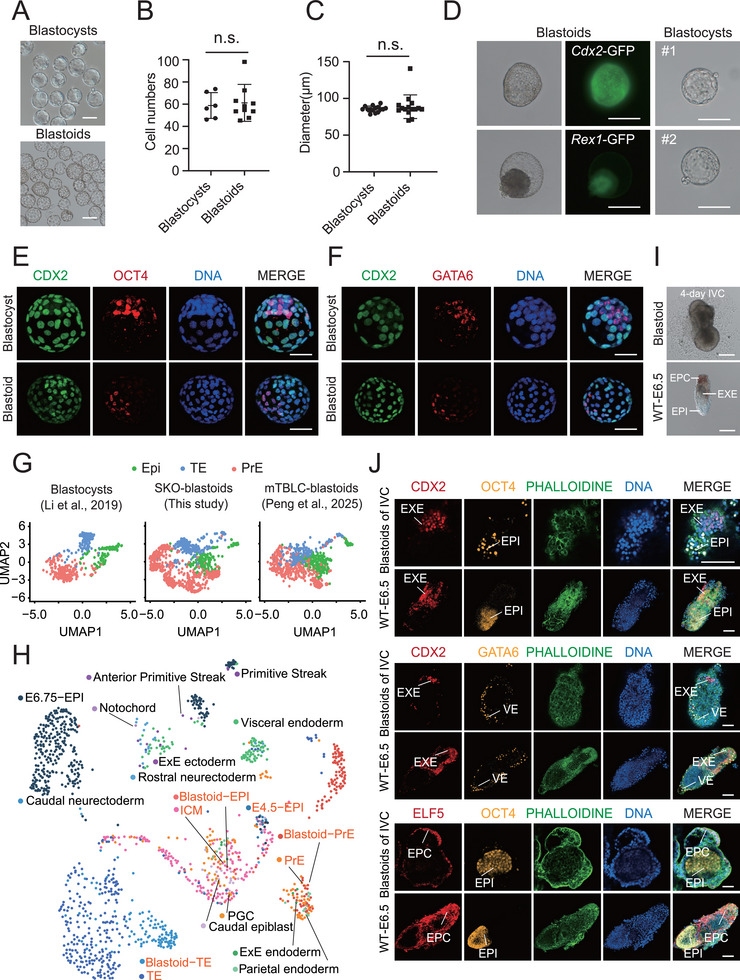
Identities and development of *Sorcs3*‐KO blastoids. A) BF images of SKO blastoids and WT blastocysts. Scale bar: 100 µm. B) The numbers of cells in SKO blastocysts were compared with those in the WT blastocysts. *t*‐test, n.s., not significant. C) The diameters of the SKO blastocysts were compared with those of the WT blastocysts. *t*‐test, n.s., not significant. D) Blastoid formation assay using *Rex1*‐GFP (RG) and *Cdx2*‐GFP (CG) SKO‐ESCs. E3.5 WT blastocysts were used as morphological references. Scale bar: 100 µm. E) IF of OCT4 (red) and CDX2 (green) in SKO blastoids. WT blastocysts were used as controls. DNA is stained with Hoechst 33342 (blue). Scale bar, 50 µm. F) IF of GATA6 (red) and CDX2 (green) in SKO blastoids. WT blastocysts were used as controls. DNA is stained with Hoechst 33342 (blue). Scale bar, 50 µm. G) UMAP projection of the single‐cell SKO blastoid, mTBLC blastoid and WT blastocyst data. H) UMAP for integration of the scRNA data of SKO blastoids and mouse embryos at different stages. I) Bright‐field images of SKO blastoids after 4 days of IVC. E6.5 WT embryos were used as a morphological reference. Scale bar: 100 µm. J) IF of CDX2 (red, EXE), OCT4 (yellow, EPI), GATA6 (yellow, VE), ELF5 (red, EPC) and PHALLOIDINE (green) of SKO blastoids subjected to IVC, with E6.5 WT embryos used as controls. Scale bar: 50 µm.

Totipotent‐like stem cells are promising sources for generating artificial blastocyst‐like structures, which are helpful for obtaining an in‐depth understanding of early embryonic development in vitro.^[^ ^9^ ^]^ Thus, we attempted to yield blastoids using presumptive totipotent SKO‐ESCs. We developed a 3D culture system in an ultralow adhesion 96‐well plate to produce blastoids according to our previous protocol^[^ ^9^ ^]^ with WT‐ESCs used as negative controls. After ≈10 days, the SKO‐ESCs produced many typical blastocyst‐like structures resembling WT blastocysts (600 SKO cells in one well formed ≈20 blastoids), but this was not observed in the WT‐ESC group (**Figure** **3**A; Figure S3A,B, Supporting Information). Next, we evaluated the quality of these blastoids in terms of cell number and diameter, with those of WT blastocysts used as references. Compared with WT blastocysts, these blastoids not only exhibited blastocyst‐like structures with visible cavities and inner cell masses (ICMs) but also had similar cell numbers and diameters (Figure 3B,C). To further assess the constitution of the blastoids, we introduced a *Rex1*‐GFP (ICM‐specific) reporter and a *Cdx2*‐GFP (TE‐specific) reporter into SKO‐ESCs separately to determine the properties of the formed blastoids. The observation of *Rex1*‐GFP‐positive and *Cdx2*‐GFP‐positive blastoids revealed their ICM and TE lineages, respectively, and were morphologically similar to those of WT blastocysts (Figure 3D). These reporter systems in the blastoids were confirmed by genotyping PCR (Figure S3C, Supporting Information). Immunofluorescence staining of OCT4 (an ICM marker), CDX2 (a TE marker), and GATA6 (a PrE marker) in blastoids confirmed their positivity for these three lineage markers (Figure 3E,F). To determine the transcriptional properties of the blastoids, we performed scRNA‐seq using 10× Genomics, the data of which were compared with published scRNA transcriptomes of mouse E3.5 WT blastocysts^[^ ^13^ ^]^ and mTBLC‐derived blastoids (a kind of published profound blastoids).^[^ ^9^ ^]^ According to the results of the comparative analysis, cells from SKO‐blastoids and mTBLC‐blastoids mostly overlapped with cells from WT‐blastocysts, suggesting that the transcriptomes of the SKO‐ and mTBLC‐blastoids were similar to those of WT‐blastocysts (Figure 3G). In accordance with certain defined specific markers of Epi, TE, and PrE, both the blastoids and blastocysts were classified into 3 subpopulations, among which they shared similar Epi, TE, and PrE clusters (Figure S3D, Supporting Information). The expression of lineage markers (Epi, *Nanog*, *Pou5f1*, *Sox2*, and *Klf2*; TE, *Cdx2*, *Gata3*, *Cdh1*, and *Krt7*; PrE, *Gata6*, *Gata4*, *Sox17*, and *Pdgfra*) confirmed that the three cell lineages existed in SKO‐blastoids and TBLC‐blastoids (Figure S3E, Supporting Information). Integration analysis revealed that the SKO‐blastoid cells resembled those of E3.5‐E4.5 WT blastocysts but not those of embryos at other stages (Figure 3H). We subsequently transferred SKO‐blastoids to the uteri of pseudopregnant mice at 2.5 days post‐coitum (dpc) and evaluated them at E6.5. The blastoids did not develop beyond E6.5 and generated deciduae only in vivo (Figure S3F, Supporting Information). Although the obtained SKO decidua was positive for CDX2, OCT4, and GATA6 (Figure S3G, Supporting Information), the failure of the blastoids to develop was consistent with the findings of other studies.^[^ ^7^, ^13^, ^14^, ^15^, ^16^, ^17^, ^18^, ^19^, ^20^ ^]^ To investigate whether these blastoids could further develop into the peri‐implantation stage, we cultured the blastoids for another 4 days in an in vitro culture (IVC) system.^[^ ^21^, ^22^ ^]^ The SKO‐blastoids attached and formed a structure similar to that of WT E6.5 embryos on Day 4 (Figure 3I), showing CDX2‐, OCT4‐, GATA6‐, and ELF5‐positive cells, which were four lineages representative of this stage (Figure 3J). Nevertheless, these results suggested that SKO‐ESCs were able to efficiently produce sound blastoids with further development potential to the peri‐implantation stage, providing an extensive source of artificial embryos for the study of early embryonic development.

### 2.4 *Tfap2c* Plays a Critical Role in the Totipotency of SKO‐ESCs

**Figure 4:**
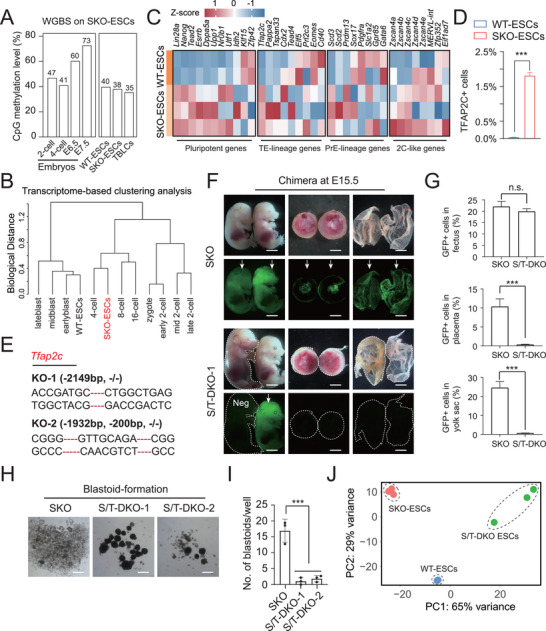
Effect of *Tfap2c*‐KO on the totipotency of SKO‐ESCs. A) Histograms showing the global CpG methylation levels of SKO‐ESCs, WT‐ESCs, TBLCs and mouse embryos based on WGBS. The published WGBS data for mouse embryos were from Wang et al. (2014), and that for TBLCs were from Shen et al. (2021). B) Transcriptome clusters of preimplantation embryos, SKO‐ESCs, and WT‐ESCs. C) Heatmap depicting the differential expression analysis between SKO‐ESCs and WT‐ESCs. D) Percentages of TFAP2C^+^ cells among the SKO‐ESC subclones and WT‐ESCs. *n* = 3; *t*‐test; ^***^ *P* < 0.001. The data are presented as the means ± SDs. E) Sanger sequencing validation of the S/T‐DKO subclones. F) E15.5 chimeric embryos derived from SKO‐ESCs and S/T‐DKO ESCs. Scale bar: 5 mm. G) GFP^+^ cell contributions to chimeric tissues: S/T‐DKO versus SKO‐ESCs. *n* = 3; *t*‐test, n.s., not significant; ^***^ *P* < 0.001. The data are presented as the means ± SDs. H) BF images of SKO blastoids and S/T‐DKO blastoids. Scale bar: 100 µm. I) The number of blastoids in each well from SKO‐ESCs and S/T‐DKO ESCs. *t*‐test, ^***^ *P* < 0.001. J) PCA of the bulk RNA‐seq data from SKO‐ESCs, WT‐ESCs, and S/T‐DKO ESCs.

To determine why SKO enhanced cell plasticity, we used whole‐genome bisulfite sequencing (WGBS) to characterize the DNA methylomes of SKO‐ESCs and WT‐ESCs. In total, we detected a global CpG methylation level of 40% in WT‐ESCs. Moreover, in SKO‐ESCs, global methylation was reduced to 38%, which was comparable to that in TBLCs (35%) (**Figure** **4**A). Next, we characterized the epigenomic characteristics using cleavage under targets and tagmentation (CUT&Tag) followed by sequencing to measure histone modifications (H3K27me3 and H3K4me3) in SKO‐ESCs and WT‐ESCs. Genome‐wide analysis revealed no obvious changes in H3K27me3 or H3K4me3 at the transcription start site (TSS) in SKO‐ESCs or WT‐ESCs (Figure S4A,B; Supporting Information). RNA analysis revealed that the global transcriptome pattern of SKO‐ESCs was similar to that of 4‐cell embryos and different from those of preimplantation embryos at other stages and WT‐ESCs (Figure 4B), indicating that SKO‐ESCs might be as totipotent as early blastomeres. Next, we compared the gene expression pattern of the SKO‐ESCs with that of the WT‐ESCs according to the bulk RNA‐seq data. The results of the heatmap analysis indicated that 2C‐specific genes (*MERVL‐int*, *Zscan4* family, and others) in SKO‐ESCs were significantly upregulated compared with their expression in WT‐ESCs. In addition to these 2C‐like‐specific genes, a TE‐specific marker, *Tfap2c*, was also upregulated in the SKO‐ESCs compared with the WT‐ESCs (Figure 4C). Thus, we performed immunofluorescence staining with antibodies against several representative TE markers (CDX2, EOMES, and TFAP2C) and PrE markers (GATA6, GATA4, and PDGFRα) in SKO‐ESCs and WT‐ESCs separately. We found that a small population of SKO‐ESCs was positive for TFAP2C, whereas other TE markers and PrE markers were not activated. Moreover, no activated TE or PrE markers were detected in WT‐ESCs (Figure S4C,D, Supporting Information). FACS analysis confirmed that TFAP2C was activated in some of the SKO‐ESCs but not in the WT‐ESCs (Figure 4D). These findings clearly indicated that SKO partially increased the expression of *Tfap2c*, which might explain the enhanced plasticity of SKO‐ESCs.

To explore whether the activation of *Tfap2c* was responsible for the totipotent‐like state of SKO‐ESCs, we devised specific Cas9‐GFP gRNAs to induce *Tfap2c*‐KO in SKO‐ESCs (Figure S4E, Supporting Information). Two *Sorcs3*/*Tfap2c*‐double‐KO (S/T‐DKO) subclones were identified through PCR and sequencing (Figure 4E; Figure S4F, Supporting Information). Furthermore, we performed chimera assays to assess totipotency. Surprisingly, S/T‐DKO‐ESCs could only integrate into the fetus and contributed little to the placenta and the yolk sac, as determined by observation and FACS analysis (Figure 4F,G; Figure S4G, Supporting Information). Next, we examined whether S/T‐DKO‐ESCs retained the capacity to spontaneously assemble into blastoids. We plated 600 S/T‐DKO‐ES cells into one well of the above‐described 96‐well plate and compared the formation efficiency and quality of the blastoids with those derived from SKO‐ESCs via the same protocol. The results indicated that S/T‐DKO‐ESCs could form only 1–2 blastoids per well, whereas SKO‐ESCs were able to yield 17 blastoids per well on average (Figure 4H,I), although there was no obvious difference in cell number or diameter between the blastoids derived from the two groups (Figure S4H,I, Supporting Information). To investigate if the reduction in the developmental potential of SKO‐ESCs was caused by *Tfap2c*‐KO, we performed bulk RNA‐seq analysis of WT‐ESCs, SKO‐ESCs, and S/T‐DKO‐ESCs. The PCA results revealed that the gene expression pattern of the S/T‐DKO‐ESCs differed from that of the SKO‐ESCs, indicating that the former no longer possessed totipotency (Figure 4J). These results suggested that the elimination of *Tfap2c* in SKO‐ESCs severely hampered totipotency, as determined by chimera assays and blastoid formation experiments.

### 2.5 Suppressing the TGF‐β, PI3K‐AKT, and Lysosome Pathways can Activate WT‐ESC Totipotency

**Figure 5:**
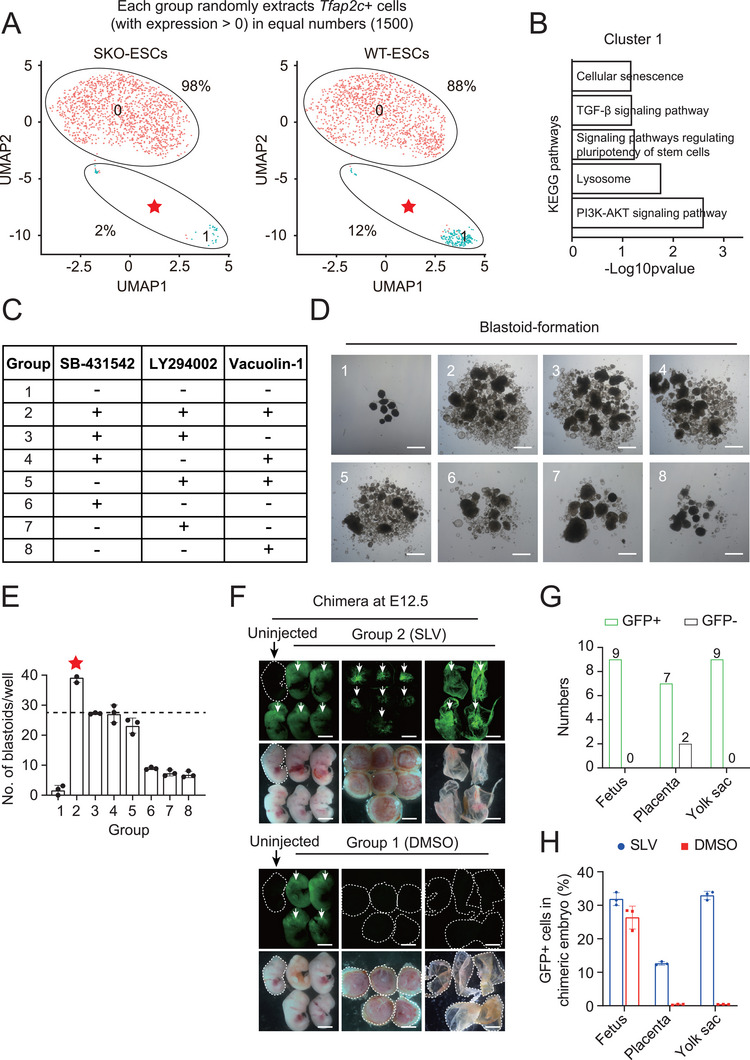
Assessment of totipotency in ESCs treated with inhibitors. A) The distribution of SKO‐ESCs and WT‐ESCs, whose datasets were extracted from equal amounts of *Tfap2c* ^+^ (expression >0) cells (1500 in each group), were compared. B) KEGG analysis of Cluster 1 SKO‐ESCs (*Tfap2c* ^+^). C) Different combinations of inhibitors used for investigating the effects of various signaling pathways on the activation of totipotency in WT‐ESCs. D) BF images of blastoids formed from WT‐ESCs after treatment with different combinations of inhibitors. Scale bar: 100 µm. E) Summary of the number of blastoids formed in each well from WT‐ESCs after treatment with different combinations of inhibitors. F) E12.5 chimeric embryos (fetuses, placentas and yolk sacs) derived from GFP‐labeled Group 2 (addition of SB‐431542, LY294002, and vacuolin‐1; SLV) ESCs and GFP‐labeled Group 1 (DMSO) ESCs, with noninjected fetuses as negative controls. Scale bar: 5 mm. G) The numbers of GFP^+^/GFP^−^ fetuses, GFP^+^/GFP^−^ placentas and GFP^+^/GFP^−^ yolk sacs in E12.5 chimeric embryos derived from GFP‐labeled SLV ESCs as donor cells. H) The percentages of GFP^+^ cells in the fetuses, placentas, and yolk sacs of chimeras derived from SLV‐ESCs and DMSO‐ESCs.

Given that SKO‐ESCs present totipotent phenotypes, comprehensively understanding the properties of SKO‐ESCs is necessary. We performed scRNA‐seq on SKO‐ESCs and their parental WT‐ESCs via 10× Genomics sequencing. Approximately 7684 SKO‐ESCs were extracted from our datasets and compared with the single‐cell transcriptomes of WT‐ESCs (13 588 cells). Dimensional reduction analyses by uniform manifold approximation and projection (UMAP) revealed that both SKO‐ESCs and WT‐ESCs could be classified into 6 distinct clusters (including Cluster 4, which expresses 2C‐representative genes, and Cluster 2, which expresses TBLC‐representative genes) and showed similar cellular compositions (Figure S5A,B, Supporting Information). The UMAP results indicated that SKO‐ESCs were heterogeneous; thus, we sought to determine the underlying mechanism of totipotency activation in SKO‐ESCs at single‐cell resolution. Since the above results confirmed that *Tfap2c* was essential for the acquisition of totipotency in SKO‐ESCs, we also found that the proportion of *Tfap2c* ^+^ cells in SKO‐ESCs was higher than that in WT‐ESCs (Figure S5C, Supporting Information). Thereafter, we extracted equal amounts of *Tfap2c* ^+^ (expression > 0) cells from among the SKO‐ESCs and WT‐ESCs (1500 each group) to analyze their differences, both of which were classified into 2 defined populations (Cluster 0 and Cluster 1). The percentage of cells in Cluster 1 among all SKO‐ESCs was ≈2%, whereas the percentage of cells in Cluster 1 among all WT‐ESCs was 12% (**Figure** **5**A). According to the results of the KEGG pathway analysis, the genes in Cluster 1 (SKO‐reduced group) were enriched mainly in the PI3K‐AKT signaling pathway, lysosomal pathway, and TGF‐β signaling pathway (Figure 5B). In addition, compared with those in WT‐ESCs, the key ligands/receptors/target genes of the TGF‐β, PI3K‐AKT, and lysosomal pathways in the SKO group were also downregulated (Figure S5D, Supporting Information). These results demonstrated that the activities of the TGF‐β, PI3K‐AKT, and lysosome signaling pathways in SKO‐ESCs clearly decreased. Next, whether a totipotent state could be activated through inhibition of these pathways was assessed. Thus, we added SB‐431542 (1 µm, an inhibitor of the TGF‐β signaling pathway; S), LY294002 (10 µm, an inhibitor of the PI3K‐AKT signaling pathway; L), and vacuolin‐1 (1 µm, an inhibitor of lysosomal catabolism; V) in different combinations to the medium to treat WT‐ESCs separately (Figure 5C). Thereafter, these groups were subjected to blastoid formation experiments to evaluate totipotency. The results revealed that any combination of the three inhibitors could produce blastoids, whereas untreated WT‐ESCs (DMSO group) did not have this ability (Figure 5D). Notably, compared with the other groups, the SB‐431542, LY294002, and Vacuolin‐1 combination group (Group 2, SLV) exhibited the highest and most repeatable blastoid formation efficiency (Figure 5E). To confirm this result, stricter chimera assays were performed. The results showed that the GFP‐ESCs in Group 2 (SLV group) could efficiently contribute to the fetus, placenta, and yolk sac of E12.5 chimeras with clear integration. However, this did not occur in the DMSO group (Figure 5F). There were a total of 9 chimeric embryos, of which 9 chimeric fetuses and 9 chimeric yolk sacs were GFP^+^ and 7 chimeric placentas were GFP^+^ (Figure 5G). FACS analysis confirmed that the GFP^+^ SLV‐ESCs contributed to ≈30.0% of the chimeric fetus, 30.0% of the chimeric yolk sac, and 12.0% of the chimeric placenta (Figure 5H). Taken together, these findings suggested that suppression of the TGF‐β, PI3K‐AKT, and lysosome signaling pathways indeed tended to increase totipotency in mouse WT‐ESCs. This provided a new strategy for developing an optimized defined medium for totipotency acquisition in mice, which would be beneficial for future research on early embryo development and the expansion of differentiation potential.

### 2.6 Characteristics of WT‐ESCs Cultured in the Defined Totipotency Medium

**Figure 6:**
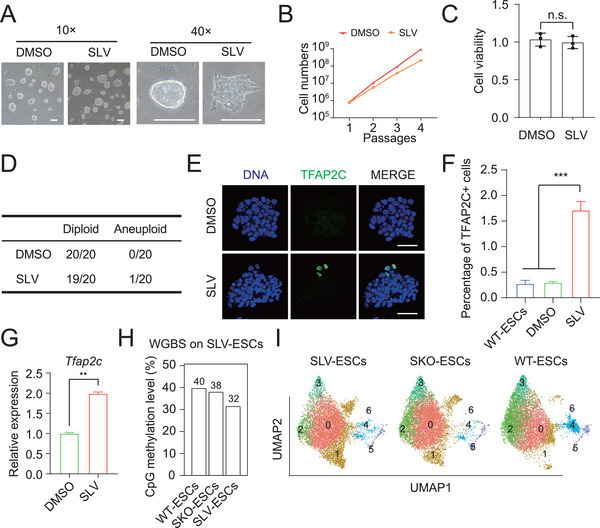
Characteristics of ESCs treated with SLV inhibitors. A) BF images of colonies of nontreated (DMSO‐) ESCs and inhibitor‐treated (SLV‐) ESCs. Scale bar: 100 µm. B) Proliferation curves of DMSO‐ESCs and SLV‐ESCs, *n* = 3. C) Assessment of the viability of DMSO‐ESCs and SLV‐ESCs in daily culture. *n* = 3; *t*‐test; n.s., not significant. The data are presented as the means ± SDs. D) Statistical analysis of chromosome spread in DMSO‐ESCs and SLV‐ESCs. E) IF of the trophoblast marker TFAP2C in DMSO‐ESCs and SLV‐ESCs. Hoechst 33342 (blue) is utilized to label DNA. Scale bar: 50 µm. F) Percentages of TFAP2C^+^ cells among DMSO‐ESCs, SLV‐ESCs, and WT‐ESCs determined by FACS analysis. *n* = 3; *t*‐test; ^***^ *P* < 0.001. The data are presented as the means ± SDs. G) Expression levels of *Tfap2c* in SLV‐ESCs and DMSO‐ESCs. H) Histograms showing the global CpG methylation levels of SLV‐ESCs, SKO‐ESCs, and WT‐ESCs. I) Comparative cell distributions among SLV‐ESCs, SKO‐ESCs, and WT‐ESCs.

To assess the basic characteristics of WT‐ESCs cultured in the above‐described optimized medium, we added inhibitors of the TGF‐β, PI3K‐AKT, and lysosome signaling pathways to ESC medium (T2iL medium described previously^[^ ^23^ ^]^) in long‐term culture. Compared with the untreated ESCs (DMSO group), the ESCs treated with the inhibitors (SLV: SB‐431542, LY294002, and vacuolin‐1) formed flatter and less compact colonies (**Figure** **6**A). We subsequently assessed the proliferation and karyotypes of the SLV‐ESCs, with DMSO‐ESCs used as controls. The results revealed that the growth of the SLV‐ESCs was slower than that of the DMSO‐ESCs (Figure 6B). Normally, ESCs in T2iL medium exhibited rapid proliferation; however, the proliferation of SLV‐ESCs was moderate and occurred at a more steady and safely sustainable pace in the presence of these inhibitors. The results of the CCK8 assays and DRAQ7 analysis indicated that SLV did not affect cell viability or induce apoptosis (Figure 6C; Figure S6A, Supporting Information). In addition, a mere karyotype abnormality was detected between the SLV‐ESCs and DMSO‐ESCs (Figure 6D; Figure S6B, Supporting Information), indicating the safety of this culture medium supplemented with SLV. Next, we performed immunofluorescence staining with antibodies against several representative stem cell markers (OCT4 and NANOG), TE markers (TFAP2C and CDX2), and PrE markers (GATA4 and GATA6) in SLV‐ESCs and DMSO‐ESCs. The number of SLV‐ESCs positive for OCT4 and NANOG did not differ from that in the DMSO group (Figure S6C, Supporting Information). Although the SLV‐ESCs were not positive for CDX2, GATA4, or GATA6 (Figure S6D,E, Supporting Information), a small population of was positive for TFAP2C (a similar phenomenon observed in SKO‐ESCs) (Figure 6E,F). Compared with that in DMSO‐ESCs, the expression of *Tfap2c* in SLV‐ESCs also differed (Figure 6G). DMSO resulted in no positive staining for any of the detected markers. We subsequently evaluated the global methylome of SLV‐ESCs by WGBS. Compared with those of WT‐ESCs and SKO‐ESCs, the global CpG methylation level of SLV‐ESCs decreased to 32%, indicating an active genome (Figure 6H). Next, we analyzed the scRNA‐seq data of SLV‐ESCs for comparison with the SKO‐ESC and WT‐ESC data (with 7500 cells randomly extracted from each group). Although they shared similar clusters, the cell cluster distribution of SLV‐ESCs was similar to that of SKO‐ESCs and distinctly different from that of WT‐ESCs (Figure 6I; Figure S6F,G, Supporting Information).

**Figure 7:**
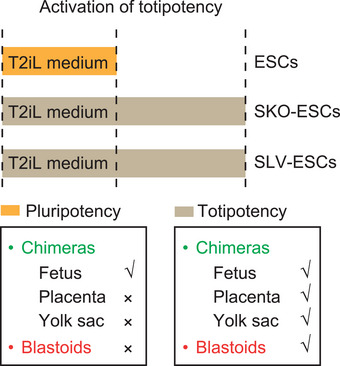
Developmental potency of SKO‐ESCs and SLV‐ESCs. A) Summary of the developmental potential of SKO‐ESCs and ESCs treated with SLV (SLV‐ESCs).

Taken together, these results suggested that SKO‐ESCs and SLV‐ESCs had full totipotent potential for both embryonic and extraembryonic (TE and PrE) lineages and could efficiently produce blastoids via safe and moderate proliferation (**Figure** **7**A).

## 3 Discussion

Two‐cell embryos are naturally totipotent and can yield embryonic and extraembryonic tissues. However, the derivation of totipotent‐like stem cells in vitro remains a great challenge. Our group has long focused on the acquisition of totipotency. We selected *Sorcs3* as a candidate restricting gene for totipotency because of its general function in receptor internalization. Whether depletion of *Sorcs3* (SKO) affects the acquisition of totipotency was unknown prior to this study. Here, we found that SORCS3 expression was very active at the morula stage (Figure S1A, Supporting Information), and this factor may be involved in cell fate segregation. As expected, SKO enabled ESCs to have the potential to differentiate into extraembryonic lineages (Figure 2), confirming that *Sorcs3* is a restrictor of totipotent reprogramming. Given that assumed totipotent‐like cells could form blastocyst‐like structures,^[^ ^7^, ^9^, ^17^, ^18^, ^20^ ^]^ we were delighted to find that SKO‐ESCs were prone to form good‐quality blastoids, which were transcriptionally similar to E3.5‐E4.5 WT blastocysts (Figure 3). This provides an easy and efficient strategy for constructing useful artificial blastoids, which is valuable for studies of early embryonic development.

TFAP2C is a member of the activating enhancer binding protein 2 (AP2) family of transcription factors,^[^ ^24^ ^]^ which are active in mouse oocytes and throughout preimplantation development (including the early ICM, TE, and subsequent trophoblast lineages).^[^ ^25^ ^]^ The overexpression (OE) of *Tfap2c* can induce trophoblast fates in mESCs.^[^ ^26^, ^27^ ^]^ Recently, TFAP2C was shown to bind both early ICM and TE genes to activate them at the totipotency stage, termed “bipotency activation”.^[^ ^28^ ^]^ Moreover, TFAP2C is a central transcription factor and has a precise regulatory effect on chromatin during murine preimplantation.^[^ ^29^, ^30^ ^]^ To this end, whether *Tfap2c* is critical for the acquisition of totipotency in SKO‐ESCs remained unclear. In our study, we found that SKO activated the expression of *Tfap2c* in some colonies, likely triggering a totipotent‐like state (Figure 4D; Figures S4C,S5C, Supporting Information). SORCS3 primarily functions through modulating receptor internalization and endosomal trafficking to control downstream signaling.^[^ ^31^ ^]^ By extension, the activation of *Tfap2c* in SKO‐ESCs might be involved in similar ways, such as through signaling pathway modulation. In WT‐ESCs, SORCS3 might promote the internalization and degradation of receptors and ligands, thereby suppressing the activation of *Tfap2c* signaling. Conversely, in SKO‐ESCs, KO of *Sorcs3* led to the deactivation of the pathways that inhibited *Tfap2c*, therefore partially activating the expression of *Tfap2c*. The deletion of *Tfap2c* in SKO‐ESCs decreased the previously gained degree of totipotency, suggesting that *Tfap2c* plays an essential role in the totipotency of SKO‐ESCs (Figure 4E–J). Moreover, we induced *Tfap2c*‐OE in WT‐ESCs and found that it could also promote totipotency to produce blastoids and efficiently contribute to the fetuses, placentas, and yolk sacs of E12.5 chimeras (Figure S7A–D, Supporting Information), further confirming the critical role of *Tfap2c*. By comparing *Tfap2c* ^+^ cells among SKO‐ESCs with those among WT‐ESCs, we found that the TGF‐β, PI3K‐AKT, and lysosomal signaling pathways in SKO‐ESCs were notably suppressed (Figure 5A,B). Furthermore, the three signaling pathways were repressed by the addition of inhibitors to the medium, which enabled WT‐ESCs to exhibit totipotency in the chimera and blastoid formation assays (Figure 5C–H). These results suggested that inhibiting these three pathways might allow the acquisition of totipotency, providing new insights into totipotency activation.

Although the crucial role of signaling pathways in cell fate determination has been recognized,^[^ ^32^, ^33^ ^]^ the impact of the coordinated inhibition of signaling pathways on totipotency activation remains largely unknown. This study was the first to demonstrate that the simultaneous suppression of the TGF‐β, PI3K‐AKT, and lysosome pathways promoted the ability of WT‐ESCs to exhibit totipotency. This discovery not only revealed a new mechanism underlying the regulation of totipotency through the modulation of critical signaling pathways but also provided a more practical method for establishing totipotent stem cell models in vitro. Mechanistically, the TGF‐β signaling pathway plays a pivotal role in cell proliferation, differentiation, and apoptosis.^[^ ^34^ ^]^ Inhibiting this pathway might disrupt original cell fate restrictions, facilitating the transition from pluripotency to totipotency. The PI3K‐AKT signaling pathway is closely associated with cell survival, proliferation, and metabolism.^[^ ^35^ ^]^ Its inhibition might modulate metabolic and growth reprogramming, creating favorable conditions for totipotency. As essential organelles within cells, lysosomes are involved in material degradation and signal regulation. A recent study revealed that inhibition of lysosomal catabolism activated a 2C‐like state^[^ ^32^ ^]^; however, the authors did not evaluate the totipotent phenotypes of the lysosome‐inactivated cells. In our assessments, compared with ESCs supplemented with the lysosome inhibitor alone, the ESCs in the medium supplemented with all three inhibitors showed definite totipotency (Figure 5E). Furthermore, long‐term culture experiments demonstrated that culture medium supplemented with the three inhibitors (SLV) had minimal effects on cell viability and genome integrity, ensuring cell safety and stability (Figure 6). To investigate whether the addition of SLV to the medium affects embryo development, we cultured fertilized 2C‐embryos in KSOM supplemented with SLV (with DMSO as a control). Surprisingly, development accelerated from the 4‐cell stage to the 8‐cell stage in the SLV group compared with the DMSO group (Figure S7E, Supporting Information). Notably, in this particular cleavage stage, the blastomeres of the embryos gradually showed developmental plasticity. However, during subsequent development, the two groups became synchronized again to the same progress at the morula stage. This characteristic gives this culture system with these three inhibitors broad prospects in future embryonic development research, regenerative medicine, and other fields.

Overall, our findings demonstrated that *Sorcs3* is a gene restricting totipotency in mESCs. Deletion of *Sorcs3* allowed mESCs to enter the TE and PrE lineages. SKO‐ESCs could also generate blastoids, whose transcriptome and peri‐implantation development potential resembled those of E3.5‐E4.5 WT blastocysts. Our results not only revealed that the degradation of *Sorcs3* followed by the activation of *Tfap2c*, could mimic the developmental process of totipotency establishment but also revealed that suppressing the TGF‐β, PI3K‐AKT, and lysosome pathways was essential for totipotency activation. In summary, this study offers a new perspective for a deeper understanding of totipotency activation and sheds light on the developmental biology of early embryogenesis.

## 4 Experimental Section

### Mice

All the mice were produced and housed at the Nankai University Animal Resources Center. Animal experiments were conducted in accordance with guidelines approved by the Animal Care and Use Committee of Nankai University (2023‐SYDWLL‐000135).

### ESC and TSC Cultures

Murine ESCs were cultured on mitomycin‐C (MCE, HY13316)‐inactivated mouse embryonic fibroblasts (MEFs) in T2iL medium.^[^ ^23^ ^]^ Mouse TSCs were maintained in TSC medium.^[^ ^4^ ^]^ All the cells were incubated at 37 °C in a humidified 5% CO_2_ atmosphere and routinely tested for mycoplasma contamination via PCR.

### Plasmid Construction and Electroporation

For Sorcs3‐KO and *Tfap2c*‐KO, single‐gRNAs (sgRNAs) were designed using the CRISPOR website (http://www.crispor.tefor.net) and cloned and inserted into the PX458 plasmid (Addgene, #48 138). For *Tfap2c*‐OE plasmid construction, the CDS of *Tfap2c* was cloned and inserted into the *PiggyBac* plasmid (SBI, PB513B). *Cdx2*‐GFP and *Rex1*‐GFP reporters were constructed according to the previous protocol.^[^ ^36^ ^]^ Approximately 8 µg of plasmid DNA was electroporated into 1×10⁶ ESCs using an electroporator (Invitrogen, Neon) under the following conditions: 1,400 V, 11 ms, and 3 pulses. To label ESCs with a β‐actin‐GFP reporter, a previously described β‐actin‐GFP donor vector and sgRNAs were used.^[^ ^37^ ^]^ The sequences of primers used in this study are listed in Table S3 (Supporting Information).

### Generation of SKO‐iTSCs, SKO‐TSCs, and SKO‐iXENs

To generate induced TSCs (iTSCs), 3×10⁴ SKO‐ESCs were trypsinized (0.05% EDTA‐trypsin; Thermo, 25300062) and plated in 6‐well plates (NEST, 703001) with TSC medium (changed every 2 days). After 7 days, CDCP1+ cells (stained with an anti‐CDCP1 antibody; R&D, AF4515) were sorted via flow cytometry (Beckman, MoFlo Astrios EQ), reseeded on MEFs, and expanded for 4–6 days with medium changes every 2 days. To generate TSCs, tetraploid embryos were generated by electrofusion (BLS, CF‐105B) of 2‐cell‐stage embryos. Following culture in KSOM (Sigma, MR‐101‐D) to the 4‐cell stage, SKO‐ESCs were injected into the embryos. The resulting chimeric blastocysts were plated in TSC medium to establish TSCs, after which the diploid cells were subsequently sorted by flow cytometry.

To induce XENs, ESCs were dissociated into single cells and plated into gelatin (Sigma, V900863) on precoated TC dishes in spontaneous differentiation medium (T2iL medium without factors).

### Mouse Chimera Assay

Chimeric embryos were generated by injecting GFP‐labeled ESCs into 4‐cell‐stage embryos (3 cells per embryo). Reconstructed embryos were cultured in KSOM for 48 h to reach the blastocyst stage. Blastocysts were then subjected to fixation, immunofluorescence staining, and imaging. Additionally, embryos were transferred to pseudopregnant mice for further analyses. Conceptuses were harvested at E6.5, E12.5, and E15.5 for imaging, immunostaining, flow cytometry, frozen sectioning, and 10× Genomics sequencing.

### Immunofluorescence Staining

Cell or embryo samples were fixed in 4% paraformaldehyde (Sigma, P6148) for 30 min at room temperature (RT), permeabilized with 0.1% Triton X‐100 (Sigma, T8787) for 1 h, and blocked with 2% BSA (Sigma, A1933) for 1 h. Primary antibodies (anti‐SORCS3 (Bioss, bs‐11503R), anti‐CDX2 (BioGenex, MU392A), anti‐EOMES (Abcam, ab183991), anti‐TFAP2C (Santa Cruz, sc‐12762), anti‐GATA6 (CST, 5851S), anti‐OCT4 (Abcam, ab81557), anti‐TPBPA (Abcam, ab104401), anti‐PROLIFERIN (Santa Cruz, sc‐271891), anti‐GCM1 (Sigma, HPA011343), anti‐SDC1 (ABclonal, a4174), anti‐GATA4 (Santa Cruz, sc‐25310), anti‐PDGFR‐α (ABclonal, A22689), and anti‐GFP (Abcam, ab13970)) were added for incubation overnight at 4 °C. After three 10‐min washes with PBS, secondary antibodies were applied for 2 h at RT. The nuclei were counterstained with Hoechst 33342 (25 µg mL^−1^; Thermo, H3570) for 15 min. Images were acquired using a Leica TCS SP8 or Zeiss LSM800 confocal microscope.

Frozen E12.5 placenta and decidua sections were stained with antibodies against TPBPA, PROLIFERIN, GCM1, TFAP2C, OCT4 (Abcam, ab181557), CDX2, and GATA6 using the aforementioned immunofluorescence protocol.^[^ ^6^ ^]^

### Generation of Blastoids

Blastoids were generated as described previously.^[^ ^13^ ^]^ Briefly, 600 WT ESCs, *Sorcs3*‐KO ESCs, *Sorcs3/Tfap2c*‐DKO ESCs, and *Tfap2c*‐OE ESCs were separately seeded in each well of ultralow adhesion 96‐well plates (Corning, 7007) with blastoid medium supplemented with 25% TSC basal medium, 25% N2B27 medium,^[^ ^38^ ^]^ 50% KSOM, 2 µm Y‐27632 (MCE, HY‐10071), 12.5 ng mL^−1^ FGF4 (MCE, HYP7014), 0.5 mg mL^−1^ heparin (MCE, HY‐17567A), 3 µm CHIR99021 (MCE, HY‐10182), 5 ng mL^−1^ BMP4 (PeproTech, 123005ET), and 0.5 µm A83‐01 (MCE, HY‐10432). Blastocyst‐like structures emerged within 8–10 days and were isolated for further self‐organization.

### IVC of Blastoids

*Sorcs3*‐KO blastoids were cultured using a published post‐implantation protocol.^[^ ^21^ ^]^ After two washes with M2 medium (Sigma, M7167), the blastoids were transferred to IVC1 medium in µ‐plates (ibidi, 80826). Upon attachment (≈2 days), the medium was exchanged for IVC2 medium for an additional 2 days of culture. The IVC1 medium contained Advanced DMEM/F12 (Thermo, 12634010) supplemented with 20% FBS, 2 mm L‐glutamine (Sigma, G8540), 1% penicillin‒streptomycin (Thermo, 15140122), 1× ITS‐X (Thermo, 12634020), 8 nm β‐mercaptoethanol (Thermo, 21985023), 200 ng mL^−1^ progesterone (MCE, HYN0437), and 25 µm N‐acetyl‐l‐cysteine (MCE, HY‐B0215). IVC2 medium contained Advanced DMEM/F12 supplemented with 30% KOSR (Thermo, 10828028), 2 mm L‐glutamine, 1% penicillin‒streptomycin, 1× ITS‐X, 8 nm β‐mercaptoethanol, 200 ng mL^−1^ progesterone, and 25 µm N‐acetyl‐l‐cysteine.

### Treatment with Signaling Pathway Inhibitors

To investigate the effects of different signaling pathways on the activation of totipotency in mESCs, a lysosomal inhibitor (1 µm vacuolin‐1, MCE, HY‐118630), a TGF‐beta pathway inhibitor (1 µm SB‐431542, MCE, HY‐10431), and a PI3K‐AKT pathway inhibitor (10 µm LY294002, MCE, HY‐10108) were added to the media of the different groups. Detains regarding the specific group allocations are described in the main text.

To determine the effects of different signaling pathways during early embryo development, fertilized 2‐cell embryos were flushed from hormone‐treated mated female mice and then cultured in KSOM for subsequent development. The embryo culture media was cultured with the SLV inhibitors (1 µm SB‐431542, 10 µm LY294002, and 1 µm vacuolin‐1), while DMSO (Sigma, D2650) was added as the blank control media. Embryonic development was observed daily, and the developmental status at each stage was analyzed.

### CCK‐8 Assays and DRAQ7 Analysis

Cell viability was determined using a Cell Counting Kit‐8 (Yeasen, 40203ES76). A total of 5 × 10^3^ cells from each sample were seeded in a well of a 96‐well plate, cultured overnight, and incubated with CCK8 for 4 h, followed by measurement with an enzyme‐labeled instrument.

Cells from each sample were resuspended in PBS to a density of 5 × 10^5^ cells mL^−1^. DRAQ7 (3 µm; MK Bio, MX4237) was added, the samples were incubated at room temperature for 15 min, and then flow cytometry analysis was performed.

### Bulk RNA‐seq, WGBS, and CUT&Tag Analyses

For bulk RNA‐seq, raw PE150 data were aligned to mm39 using STAR 2.5.3. Read counts were generated with featureCounts, followed by DESeq2 differential expression analysis (|fold change| >2, adjusted *p* <0.05) for heatmap construction and PCA.

For WGBS, paired‐end reads were trimmed by the Trim Galore software with the default parameters. The cleaned data were aligned to the mm39 reference in Bismark/Bowtie2 mode. Duplicate reads were removed by the sambamba markdup command, and the methylation calls were extracted from unique alignment using bismark_methylation_extractor with the “–bedGraph” parameter. The DNA methylation data of embryo cells preimplantation were downloaded from GSE56697.^[^ ^39^ ^]^

The CUT&Tag assay was performed using a Hyperactive Universal CUT&Tag Assay Kit from Illumina (Vazyme, TD904‐01) following the manufacturer's instructions. An Illumina NovaSeq 150PE platform was used for sequencing. Paired‐end sequencing reads were trimmed with the Trim Galore software with the default parameters. After trimming, the reads were aligned to the reference mouse mm39 assembly using Bowtie2. The resulting alignments, recorded in a BAM file, were sorted, indexed, and marked for duplicates with the Piccard (http://broadinstitute.github.io/picard) MarkDuplicate function. The BAM file was filtered with SAMtools to discard certain reads. MACS2 was subsequently used to call peaks from the BAM file.

### Analysis of the Single Cell RNA‐Seq Data

Cells were prepared by washing and resuspended in 1× PBS (calcium and magnesium‐free) supplemented with 0.04% BSA. Approximately 10000 viable cells were loaded onto a Chromium Single‐Cell Controller to generate Gel Bead‐in‐Emulsions (GEMs) containing cDNA. The recovered cDNAs were used to construct libraries using the Single‐Cell 3′ Library and Gel Bead Kit V3 (10x Genomics, PN‐1000075) according to the manufacturer's instructions. The scRNA‐seq data for the SKO‐ESCs, WT‐ESCs, and SLV‐ESCs were processed, aligned, and quantified using the cellranger count pipeline (version 8.0.0), and the reads were mapped to the mm10 reference genome. Gene expression in single cells from different samples was merged using the Seurat R package 5.0.3.^[^ ^40^ ^]^ Cell clustering and UMAP visualization were performed using the FindClusters and RunUMAP functions.

As shown in Figure 5A, equal amounts of *Tfap2c*+ (expression >0) SKO‐ESCs and WT‐ESCs (1500 each group) were used for the difference analysis. For blastoids, cell clusters were identified on the basis of gene signatures calculated with the FindAllMarkers function. For an integrated analysis with mouse embryo data, 1000 blastoid cells were sampled. Blastocyst scRNA‐seq data were downloaded from the GEO database (GSE135701). To eliminate batch effects, the E3.5 blastocyst dataset^[^ ^13^ ^]^is integrated with FindIntegrationAnchors and IntergrateData.

### Statistical Analyses and Reproducibility

The experiments in this study were performed with at least three biological replicates unless otherwise specified. All the statistical analyses were performed with GraphPad Prism 8.0 software. The statistical tests used are listed in the figures and figure legends. Details of the statistical tests are outlined in the figure legends. ^*^ *P* < 0.05, ^**^ *P* < 0.01, ^***^ *P* < 0.001.

## Conflict of Interest

The authors declare no conflict of interest.

## Author Contributions

W.Z., X.M., and Y.H. contributed equally to this work. L.S. and Q.G. designed and supervised the study. W.Z., X.M., and Y.H. performed most of the experiments. W.Z. analyzed the bioinformatics data. Q.J., Y.Z., X.D., X.L., S.S., X.S., and D.D. participated in the cell culture and molecular experiments. W.Z., Y.H., Y.S., Q.G. and L.S. wrote the manuscript draft. Q.G. and L.S. verified the final manuscript.

## Supporting information



Supporting Information

Supplemental Data 1

## Data Availability

The data underlying this article are available in the article and in its online supplementary material. The raw dataset in this study have been deposited in the Genome Sequence Archive of the Beijing Institute of Genomics (BIG) Data Center with accession numbers CRA026477.
