# Supplementary material for: Depletion of Sorcs3 Activates Totipotency in Mouse Embryonic Stem Cells by Modulating Key Signaling Pathways
Source: Adv Sci (Weinh). 2025 Nov 3;13(7):e09151. doi: 10.1002/advs.202509151 (PMC12866853; doi:10.1002/advs.202509151)
Supplement: Supplementary file 1 — Supporting Information [file ADVS-13-e09151-s001.docx]

**SUPPORTING information**

**Title:** Depletion of *Sorcs3* activates totipotency in mouse embryonic stem cells by modulating key signaling pathways

**Running title:** A profound cocktail medium for totipotency

**Authors and affiliations:**

Wenhao Zhang^1,3^, Xinyu Mao^1,3^, Yu He^2,3^, Qingshen Jia^1^, Yiding Zhao^1^, Xiaomeng Dai^1^, Xiaoyan Li^1^, Shengyi Sun^1^, Xiaoyan Sheng^1^, Dan Ding^1^, Yuan Shi^3,*^, Qian Gao^1,*^ and Ling Shuai^1,2,*^

1 State Key Laboratory of Medicinal Chemical Biology, College of Pharmacy, Nankai Animal University Resources Center and Reproductive Regulation and Institute of Transplantation Medicine, Nankai University, Tianjin 300350, China.

2 Department of Neonatology, Children’s Hospital of Chongqing Medical University, National Clinical Research Center for Child Health and Disorders, Ministry of Education Key Laboratory of Child Development and Disorders, China International Science and Technology Cooperation Base of Child Development and Critical Disorders, Chongqing Key Laboratory of Child Rare Diseases in Infection and Immunity, Chongqing, 400014, China.

3 These authors contributed equally to this work.

*** Correspondences:** shiyuan@hospital.cqmu.edu.cn (Y.S.); gaoqian@nankai.edu.cn (Q.G.); lshuai@nankai.edu.cn (L.S.)

**Lead Contact:** lshuai@nankai.edu.cn (L.S.)


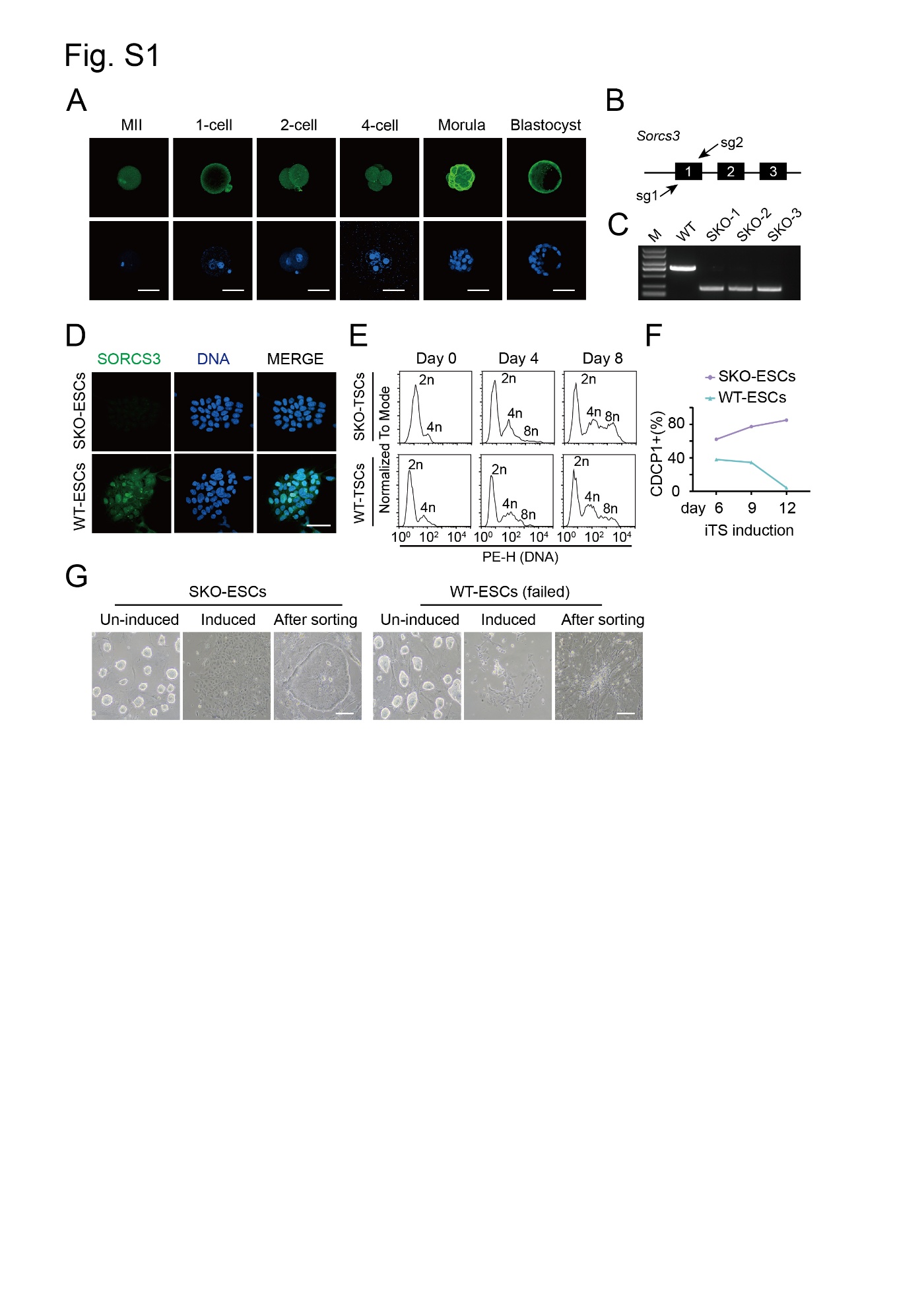


**Figure S1. Induction of TSCs using SKO-ESCs**

1. Immunofluorescence (IF) images of SORCS3 (green) in different stages of pre-implantation embryos. Hoechst 33342 (blue) is utilized to label nuclei. Scale bar: 50 μm.
2. Schematic design for *Sorcs3* gene knock-out (KO) vectors.
3. Genotyping PCR results of *Sorcs3*-KO (SKO) subclones.
4. IF images of SORCS3 (green) in SKO-ESCs and WT-ESCs. Hoechst 33342 (blue) is utilized to label nuclei. Scale bar: 50 μm.
5. DNA content analysis of differentiated cells from TSCs (SKO- and WT-) during spontaneous differentiation on Day 0, 4, and 8. The DNA contents of diploid (2n), tetraploid (4n), and octoploid (8n) are indicated.
6. Flow cytometric analysis of CDCP1^+^ cell proportions during *in vitro* conversion of TSC using SKO-ESCs and WT-ESCs (on Day 6, 9, 12), respectively.
7. Morphological comparison of iTSCs derived from SKO-ESCs and those from WT-ESCs under identical conditions after cell sorting enrichment with the CDCP1-antibody in bright field (BF). Scale bar: 100 μm.


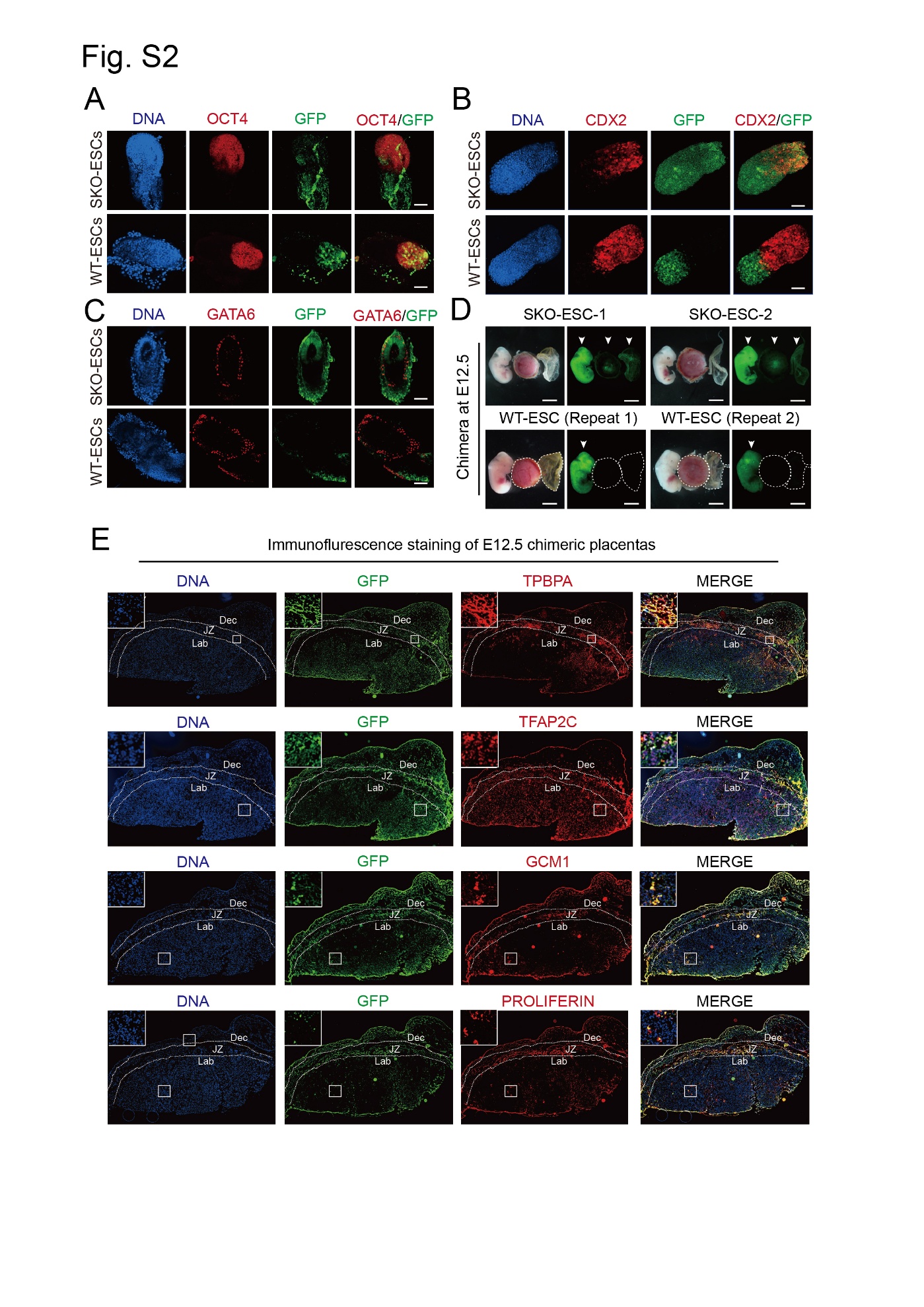


**Figure S2. SKO-ESCs could differentiate to extraembryonic lineages *in vivo***

1. IF images of E6.5 chimeric embryos injected with SKO-ESCs (green) and WT-ESCs (green) separately, being stained with OCT4 (red) and Hoechst 33342 (blue). Scale bar, 50 μm.
2. IF images of E6.5 chimeric embryos injected with SKO-ESCs (green) and WT-ESCs (green) separately, being stained with CDX2 (red) and Hoechst 33342 (blue). Scale bar, 50 μm.
3. IF images of E6.5 chimeric embryos injected with SKO-ESCs (green) and WT-ESCs (green) separately, being stained with GATA6 (red) and Hoechst 33342 (blue). Scale bar, 50 μm.
4. E12.5 chimeric fetuses, placentas and yolk sacs derived from GFP-labeled ESCs (SKO- and WT-). Scale bar: 5 mm.
5. IF sections of E12.5 chimeric placentas from GFP-labeled SKO-ESCs (green), being co-stained with trophoblast markers (TPBPA, TFAP2C, GCM1, PROLIFERIN; red) and Hoechst 33342 (blue). Scale bar: 50 μm.


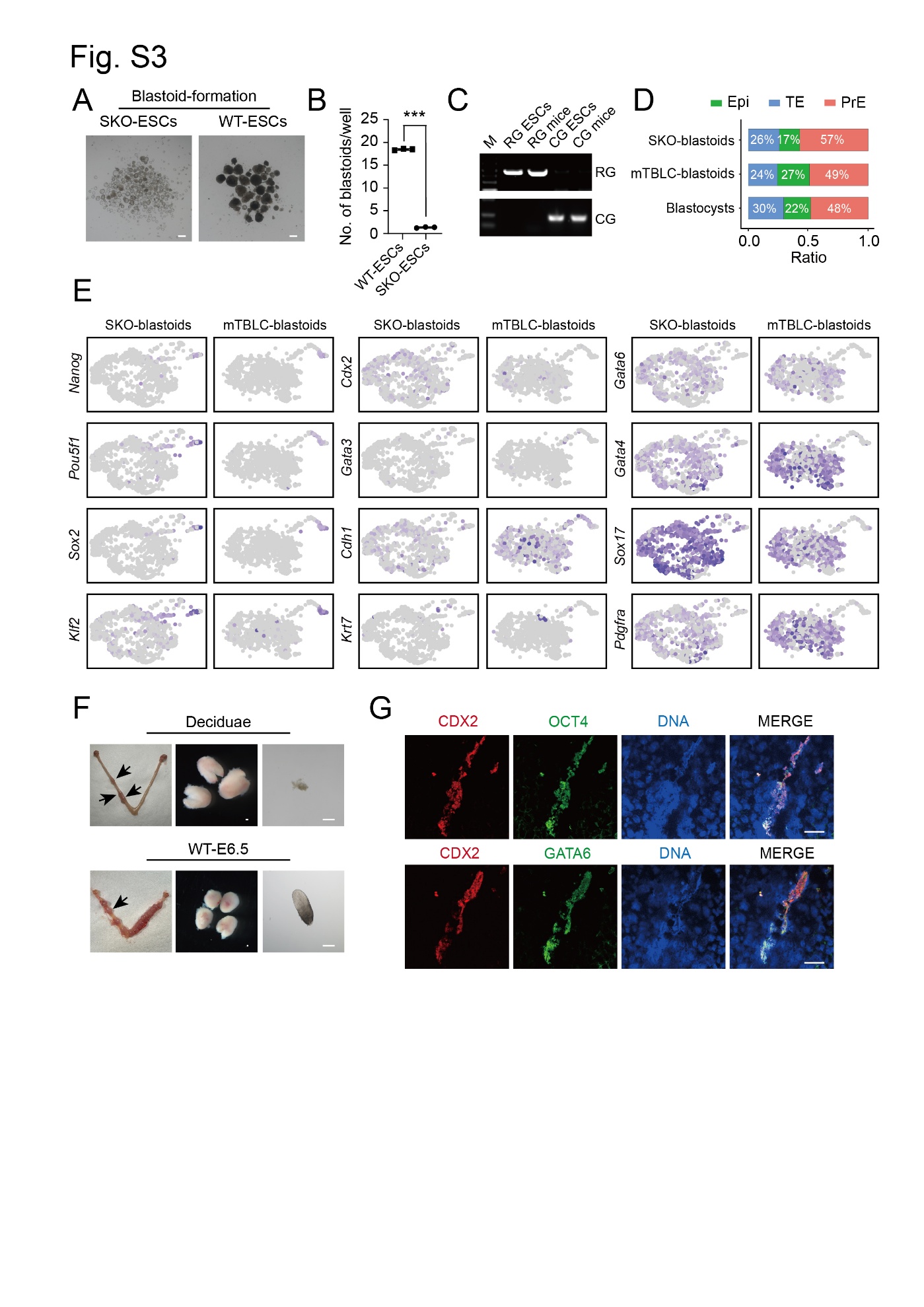


**Figure S3. Characteristics of SKO-blastoids**

1. BF images of SKO-ESCs derived blastoids and WT-ESCs derived aggregates. Scale bar: 100 μm.
2. The numbers of blastoids derived from ESCs (SKO- and WT-) in each well. *n* = 3, *t*-test, ****P* < 0.001.
3. Genotyping of *Rex1*-GFP (RG) and *Cdx2*-GFP (CG) reporters in SKO-ESCs. RG and CG mice tail-tip are used as positive controls.
4. Percentages of cells in the Epi, TE, and PrE clusters of SKO-blastoids, mTBLC-blastoids, and WT-blastocysts, respectively.
5. Single-cell expression profiles of lineage specific markers: Epi, *Nanog*, *Pou5f1*, *Sox2*, and *Klf2*; TE, *Cdx2*, *Gata3*, *Cdh1*, and *Krt7*; PrE, *Gata6*, *Gata4*, *Sox17*, and *Pdgfra* in SKO-blastoids and mTBLC-blastoids.
6. E6.5 deciduae-like structures derived from transferred SKO-blastoids compared with WT-E6.5 embryos. Scale bar: 100 μm.
7. IF images of TE marker CDX2 (red), Epi marker OCT4 (green), and visceral endoderm (VE) marker GATA6 (green) in E6.5 deciduate-like structures derived from SKO-blastoids. Scale bar: 50 μm.


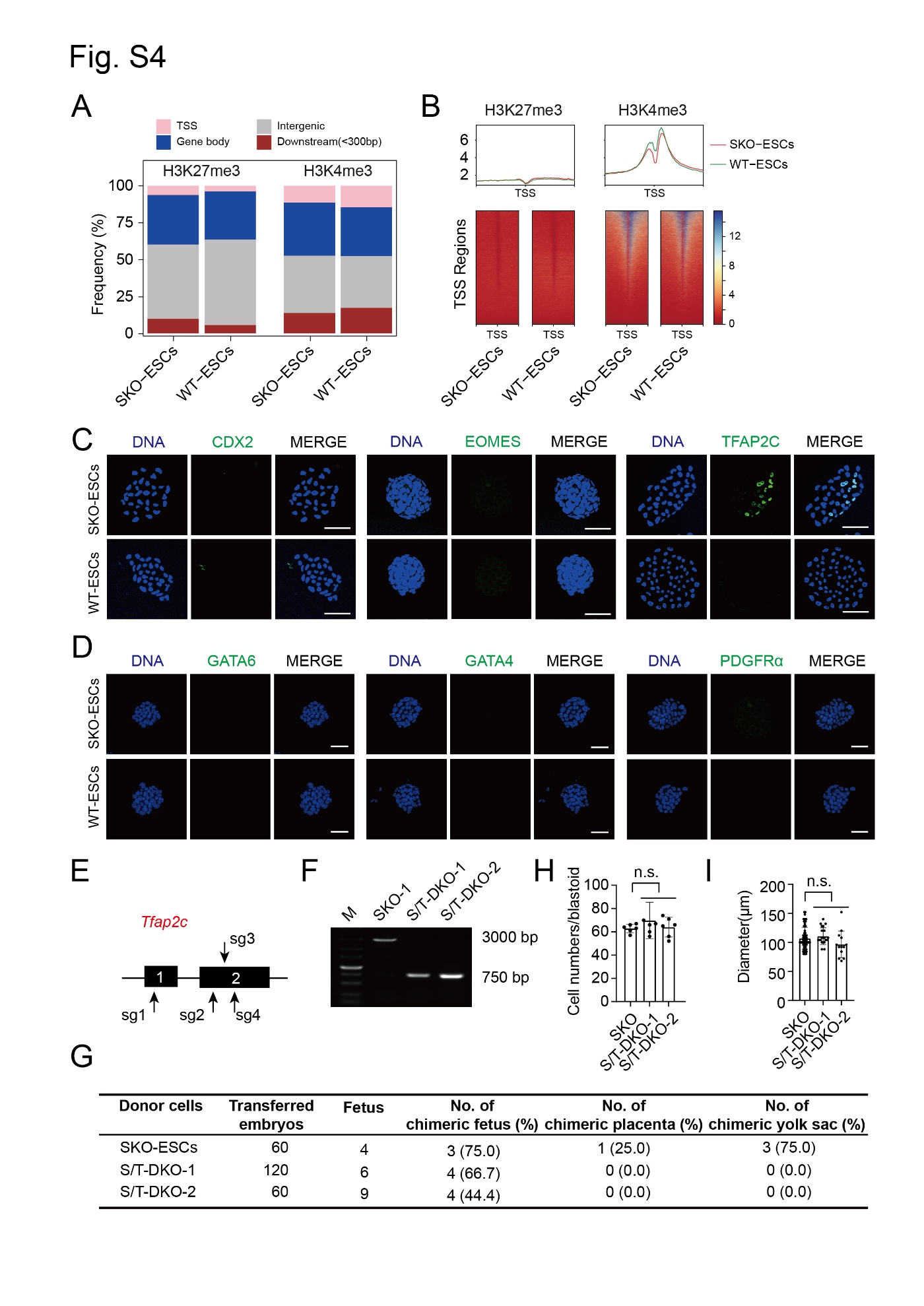


**Figure S4. The activation of *Tfap2c* in partial SKO-ESCs**

1. Bar graphs display the genome-wide distributions of H3K27me3 and H3K4me3 peaks in SKO-ESCs and WT-ESCs.
2. Average signals of H3K27me3 and H3K4me3 of all RefSeq genes in SKO-ESCs and WT-ESCs. TSS: transcription starting site.
3. IF images of TE markers (CDX2, EOMES, and TFAP2C) in SKO-ESCs and WT-ESCs, independently. Hoechst 33342 (blue) is utilized to label nuclei. Scale bar: 50 μm.
4. IF images of PrE markers (GATA6, GATA4, and PDGFRα) in SKO-ESCs and WT-ESCs, independently. Hoechst 33342 (blue) is utilized to label nuclei. Scale bar: 50 μm.
5. Design diagram of the *Tfap2c* gene KO vectors.
6. Genotyping PCR results of *Tfap2c* in SKO-ESCs and S/T-DKO (*Sorcs3* and *Tfap2c* double KO) subclones.
7. Chimeric contribution efficiencies of SKO-ESCs and S/T-DKO ESCs at E15.5, separately.
8. The cell numbers of S/T-DKO blastoids, compared with those of SKO blastoids. *t*-test, n.s., not significant.
9. The diameters of S/T-DKO blastoids, compared with those of SKO blastoids. *t*-test, n.s., not significant.


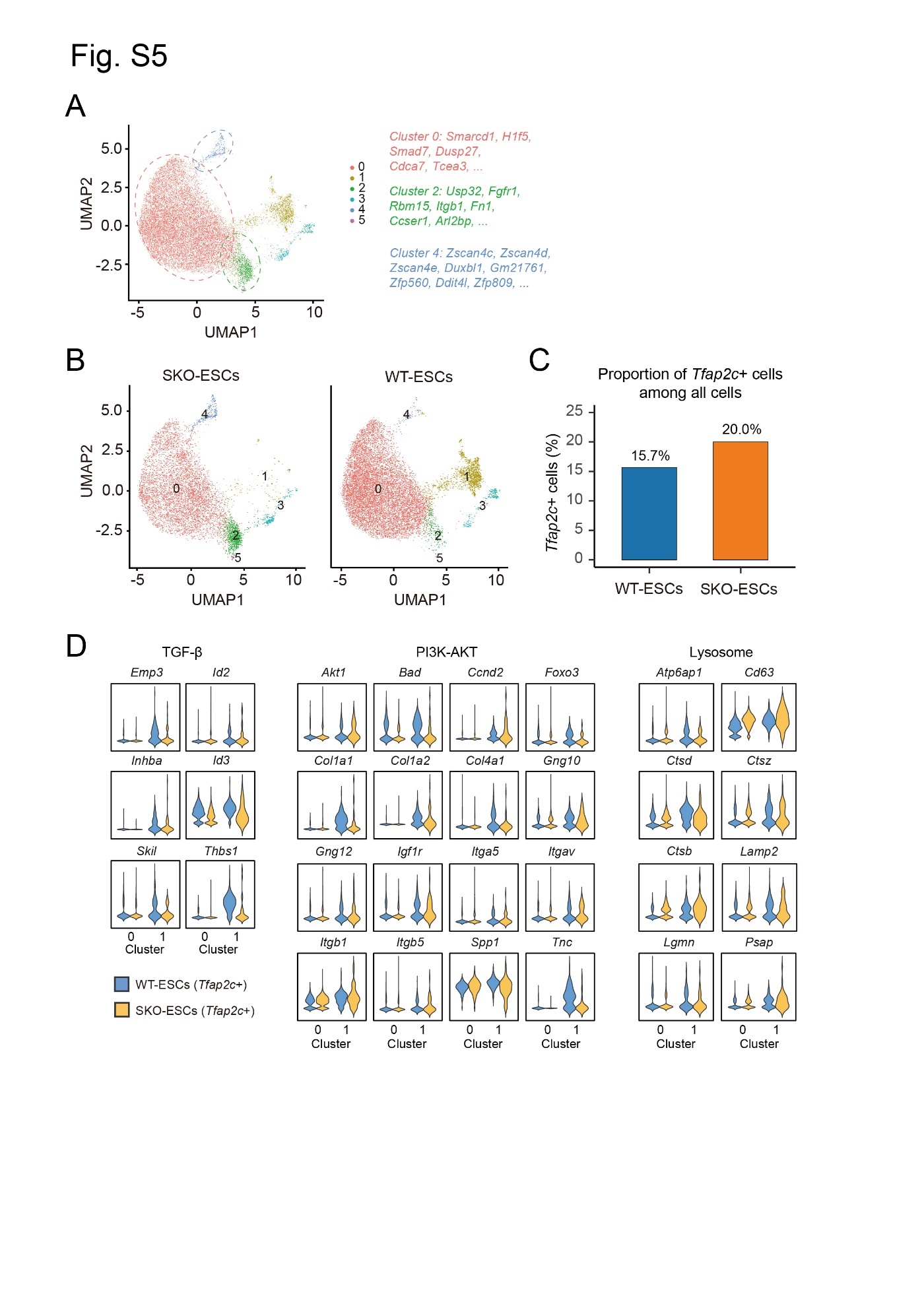


**Figure S5. Single-cell RNA-seq analysis of SKO-ESCs**

1. UMAP visualization of single-cell clusters in SKO-ESCs and WT-ESCs. Shown are some representative genes in each cluster.
2. Comparative cell distribution in SKO-ESCs and WT-ESCs.
3. Proportion of *Tfap2c*^+^ cells in SKO-ESCs and WT-ESCs.
4. Violin plot of the ligands/receptors/target genes of TGF-β, PI3K-AKT, and lysosomal pathways in *Tfap2c*^+^ cells in SKO-ESCs and WT-ESCs.


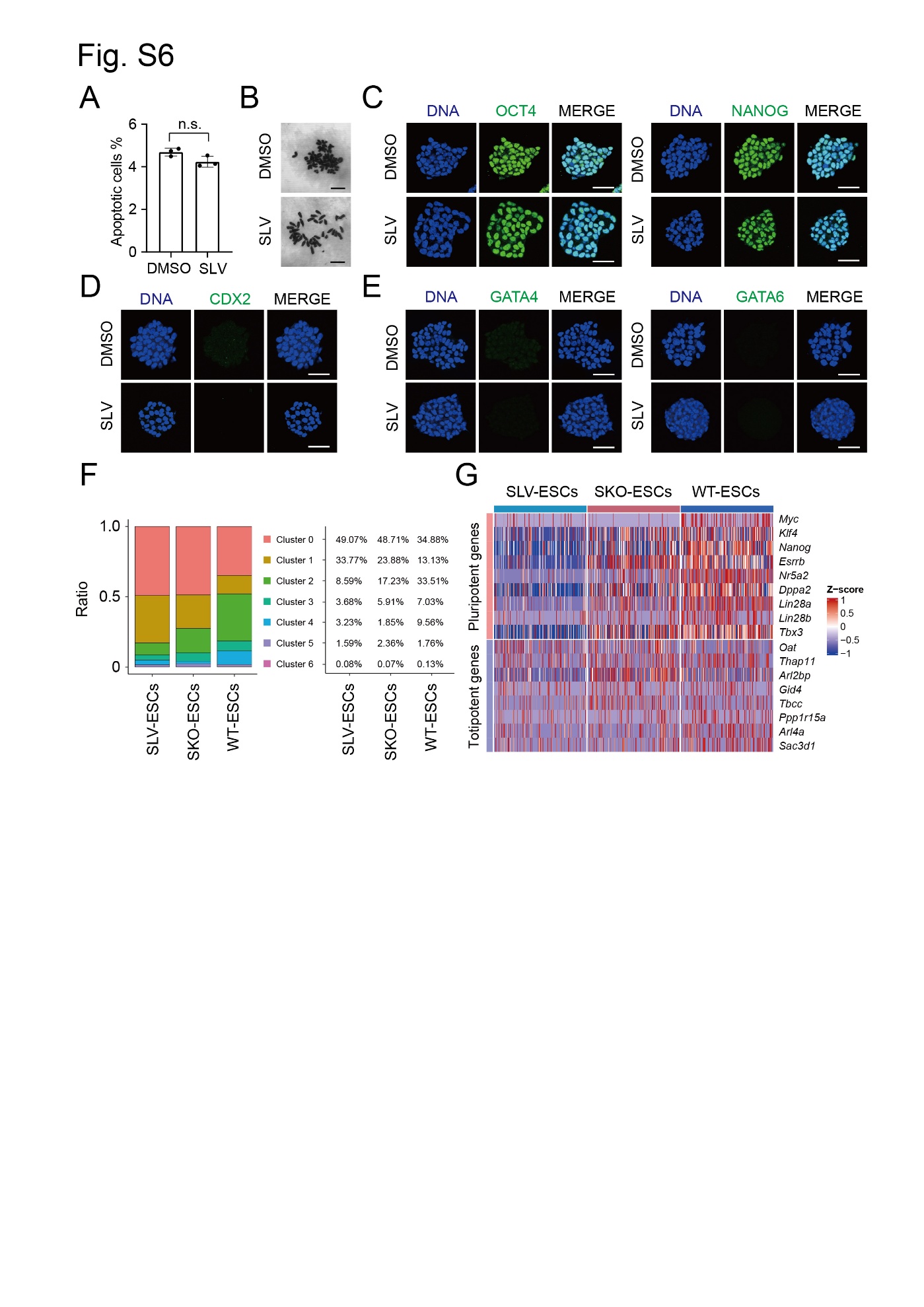


**Figure S6. Characteristics of SLV-ESCs**

1. Apoptosis analysis of DMSO group and SLV group by DRAQ7 staining. *n* = 3, *t*-test, n.s., no significant.
2. Chromosome spread analysis of DMSO group and SLV group. Scale bars, 10 μm.
3. IF images of pluripotent markers (OCT4 and NANOG) in DMSO group and SLV group. Hoechst 33342 (blue) is utilized to label nuclei. Scale bar: 50 μm.
4. IF images of TE marker (CDX2) in DMSO group and SLV group. Hoechst 33342 (blue) is utilized to label nuclei. Scale bar: 50 μm.
5. IF images of PrE markers (GATA4 and GATA6) in DMSO group and SLV group. Hoechst 33342 (blue) is utilized to label nuclei. Scale bar: 50 μm.
6. Percentages of cells in clusters of SLV-ESCs, SKO-ESCs, and WT-ESCs, respectively.
7. Heatmap of pluripotent and totipotent genes from scRNA-seq data of SLV-ESCs, SKO-ESCs, and WT-ESCs.


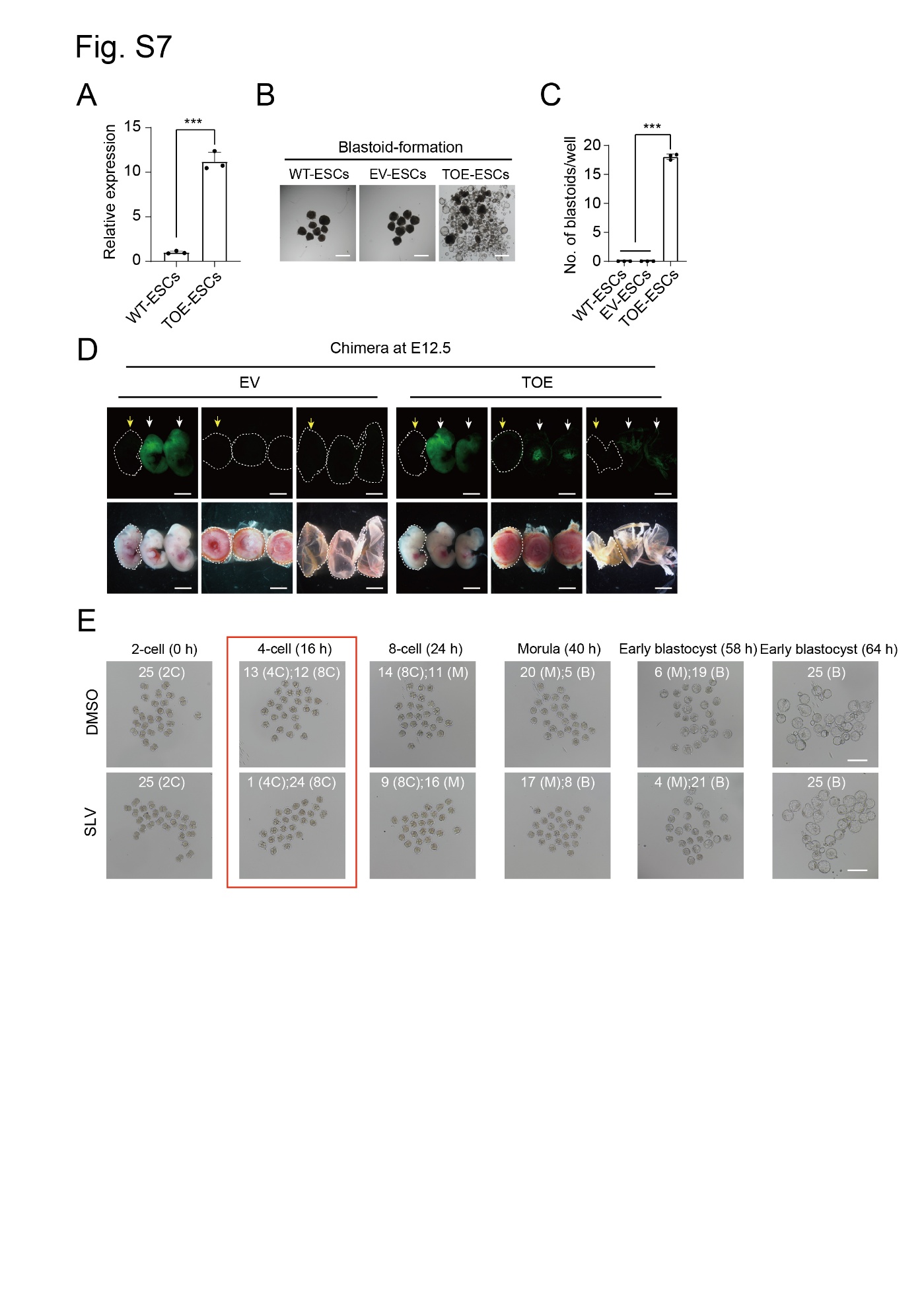


**Figure S7. Effect of SLV addition on development**

1. The expression levels of *Tfap2c* in WT-ESCs and *Tfap2c*-OE-ESCs.
2. BF images of blastoids produced from WT-ESCs, EV-ESCs, and TOE-ESCs. Scale bar: 100 μm.
3. The summary of blastoid-formed numbers of WT-ESCs, EV-ESCs and TOE-ESCs in each well, separately.
4. E12.5 chimeric embryos (fetus, placenta and yolk sac) derived from GFP-labeled EV-ESCs and GFP-labeled TOE-ESCs. White arrow indicates the chimeric fetuses, placentas, and yolk sacs. Yellow arrow indicates the un-injected ones as negative controls. Scale bar: 5 mm.
5. The development of the pre-implantation embryos with SLV and without SLV (DMSO) in the medium. Scale bar: 100 μm.

**Table S1. Contribution of SKO-ESCs to chimeric fetus, placentas, and yolk sacs.**

| Development state | Donor cells | No. of transferred embryos (%) | No. of embryos (%) ^a^ | No. of chimeric fetus (%) ^b^ | No. of chimeric placenta (%) ^c^ | No. of chimeric yolk sac (%) ^d^ |
| --- | --- | --- | --- | --- | --- | --- |
| E12.5 | SKO-ESC-1 | 87 | 14 (16.1) | 10 (71.4) | 8 (57.1) | 9 (64.3) |
|  | SKO-ESC-2 | 90 | 13 (14.4) | 10 (76.9) | 9 (69.2) | 9 (69.2) |
|  | WT-ESCs | 86 | 10 (11.6) | 8 (80.0) | 0 (0.0) | 0 (0.0) |
| E15.5 | SKO-ESC-1 | 94 | 10 (10.6) | 7 (70.0) | 6 (60.0) | 6 (60.0) |
|  | SKO-ESC-2 | 92 | 10 (10.9) | 8 (80.0) | 7 (70.0) | 7 (70.0) |
|  | WT-ESCs | 89 | 11 (12.4) | 7 (63.6) | 0 (0.0) | 0 (0.0) |

1. Percentage is calculated based on the number of transferred embryos;
2. Percentage is calculated based on the number of dissected embryos;
3. Percentage is calculated based on the number of dissected placentae;
4. Percentage is calculated based on the number of dissected yolk sac.

**Table S2. Primer sequences.**

| *Sorcs3*-KO | *Sorcs3* sg1-1 | caccgCCATCGGACCCGCACCTCGG |
| --- | --- | --- |
|  | *Sorcs3* sg1-2 | aaacCCGAGGTGCGGGTCCGATGGc |
|  | *Sorcs3* sg2-1 | caccgTCACACCAAGGGTTCGCGAG |
|  | *Sorcs3* sg2-2 | aaacCTCGCGAACCCTTGGTGTGAc |
| *Tfap2c*-KO | *Tfap2c* sg1-1 | caccgCCGATGCGCGTCCAGTGACT |
|  | *Tfap2c* sg1-2 | aaacAGTCACTGGACGCGCATCGGc |
|  | *Tfap2c* sg2-1 | caccgCCCGGCGGGAAGTCTACCGC |
|  | *Tfap2c* sg2-2 | aaacGCGGTAGACTTCCCGCCGGGc |
|  | *Tfap2c* sg3-1 | caccgCGCCGACCATTACTCGCATC |
|  | *Tfap2c* sg3-2 | aaacGATGCGAGTAATGGTCGGCGc |
|  | *Tfap2c* sg4-1 | caccgGCGCCGCCTCTCTCGCACAC |
|  | *Tfap2c* sg4-2 | aaacGTGTGCGAGAGAGGCGGCGCc |
| Genotype | *Sorcs3* genotype F | TCCTCATTCCCCTGACACACT |
|  | *Sorcs3* genotype R | ACACTGCTGTTGTGTCCCGA |
|  | *Tfap2c* genotype F | GATGAAGGACGGTTCTTGAGGT |
|  | *Tfap2c* genotype R | GTGAGCCGTGTTGGCCACTTA |
| qPCR | *Gapdh* F | AGGTCGGTGTGAACGGATTTG |
|  | *Gapdh* R | TGTAGACCATGTAGTTGAGGTCA |
|  | *Tfap2c* F | ATCCCTCACCTCTCCTCTCC |
|  | *Tfap2c* R | CCAGATGCGAGTAATGGTCGG |
